# Effects of Bt Rice Straw Leachates on *Daphnia magna*

**DOI:** 10.3390/ijms27219962

**Published:** 2026-11-06

**Authors:** Congyu Zhang, Jiahang Liang, Xueli Zhang, Lipeng Zhao, Yifeng Jiang, Huizhen Fu, Wanting Deng, Yuanjiao Feng

**Affiliations:** 1 Key Laboratory of Agro-Environment in the Tropics, Ministry of Agriculture and Rural Affairs, College of Resources and Environment, South China Agricultural University, Guangzhou 510642, China; cyzhang1030@163.com (C.Z.); 13822612635@163.com (J.L.); 13610202007@139.com (X.Z.); tdftrdhth@163.com (L.Z.); jiangyf0617@163.com (Y.J.); 13827861641@163.com (H.F.); 15622507300@163.com (W.D.); 2 Guangdong Engineering Research Center for Modern Eco-Agriculture and Circular Agriculture, Guangzhou 510642, China; 3 Department of Ecology, College of Natural Resources and Environment, South China Agricultural University, Guangzhou 510642, China

**Keywords:** Bt rice, *Daphnia magna*, antioxidant enzyme activity, malondialdehyde

## Abstract

Cry1Ab proteins derived from *Bacillus thuringiensis* (Bt) rice can be released into paddy water through straw leaching. Straw from non-Bt rice (Tianyou Huazhan), homozygous Bt rice (T775), and heterozygous Bt rice (T775 F_1_ hybrid) was used to expose *Daphnia magna* over two consecutive generations, each for 21 days, at nominal straw-loading concentrations of 1, 2, 4, and 8 g L^−1^. Survival, growth, reproduction, catalase (CAT), peroxidase (POD), superoxide dismutase (SOD), and malondialdehyde (MDA) were evaluated. Growth and reproductive responses were described using originally reported means, standard errors, and Duncan groupings, while traceable biochemical measurements underwent generation-specific two-way analysis of variance and prespecified control comparisons. Changes in individual and biochemical endpoints varied by rice type, loading concentration, and generation, without a consistent monotonic concentration–response relationship. Fisher’s exact tests showed no significant differences in 21-day survival between either Bt treatment and the corresponding non-Bt treatment at the same concentration. Changes in enzyme activities and MDA alone do not establish oxidative injury, and descriptive differences between generations cannot establish cumulative toxicity.

## 1. Introduction

Rice is an important food crop, and insect-pest management is closely linked to sustainable rice production. Transgenic rice expressing insecticidal proteins derived from *Bacillus thuringiensis* (Bt) offers an additional approach to controlling certain lepidopteran pests [1,2]. However, residues remaining after Bt-crop cultivation and their released constituents may enter adjacent aquatic environments, highlighting the need to assess exposure and responses in non-target aquatic organisms [3,4].

Paddy fields are both agricultural production systems and shallow-water ecosystems. When Bt rice straw remains in the field or is returned to the soil, leaching and decomposition may release its constituents into the water; low concentrations of Cry proteins have been detected in paddy water [5,6]. Unlike purified Cry-protein exposure, straw leachates may also contain dissolved organic matter, nutrients, and other plant-derived constituents. Consequently, observed biological responses may be related to Cry protein, the straw matrix, or both. Differences between Bt and non-Bt treatments alone cannot identify the causative constituent [7,8].

*Daphnia magna* is a model planktonic crustacean commonly used in aquatic ecotoxicology. Its survival, growth, reproduction, and antioxidant-related physiological endpoints can be used to characterize organism-level responses under laboratory exposure. Previous laboratory studies and field surveys have examined the effects of Bt-crop residues and purified proteins on aquatic organisms, although the test materials, exposure routes, and endpoints vary [3,9,10,11]. For example, certain transgenic-maize materials did not cause adverse growth or reproductive responses in laboratory *D. magna* assays [12], while surveys of paddy-field zooplankton did not consistently identify significant differences in community metrics [13,14]. These findings underscore the need to evaluate each exposure system and crop material separately; evidence from other crops cannot be directly extrapolated to Bt rice straw leachates [15].

Rice straw leaching and decomposition may alter the composition of dissolved substances in surrounding water [8]. Bt crops and their non-transgenic comparison materials may differ in genetic background, nutrient composition, or secondary metabolites. Such potential confounding factors should therefore be considered when interpreting the ecotoxicological responses to Bt materials [7]. Studies of microbial communities associated with Bt-rice cultivation provide additional environmental context [16], but findings from other crops or exposure systems cannot replace direct tests of the rice straw leachates examined here.

Although previous research has informed the assessment of Bt crops in non-target aquatic organisms, several questions remain for Cry1Ab-expressing rice: whether homozygous Bt, heterozygous Bt, and non-Bt straw leachates produce different growth, reproductive, and antioxidant-related responses at the same nominal straw loading; whether these responses vary consistently with loading concentration; and how observations compare across two successive generations. Studies of purified Cry proteins, other crop materials, or different contaminants provide comparative context but cannot substitute for direct evaluation of the present leachate system [17,18,19,20]. Because generation-related interactions were not evaluated in a joint model, cumulative transgenerational toxicity was not assumed.

Accordingly, straw leachates from non-Bt Tianyou Huazhan rice, homozygous Bt T775 rice expressing Cry1Ab, and heterozygous Bt T775 F_1_ rice were prepared at nominal straw mass concentrations of 1, 2, 4, and 8 g L^−1^. *Daphnia magna* was exposed for 21 days in two successive generations. Survival, growth, reproduction, CAT, POD, and SOD activities, and MDA content were examined to describe organism-level responses to different rice-straw types and leachate loadings, providing a basis for future work separating Cry-protein effects from matrix effects and for field validation.

## 2. Results

### 2.1. *Effects of Bt Rice Straw Leachates on First-Generation *Daphnia magna

#### 2.1.1. Cry1Ab Concentrations in First-Generation *D. magna*

After 21 days of exposure, Cry1Ab was quantified in first-generation *D. magna* samples by enzyme-linked immunosorbent assay (ELISA) (Table 1). The highest measured concentrations within both the homozygous- and heterozygous-Bt groups occurred at 8 g L^−1^. At the same nominal straw concentration, values were generally higher in the homozygous-Bt group than in the heterozygous-Bt group.

#### 2.1.2. Effects on Growth and Reproductive Parameters

Growth parameters of first-generation *D. magna* are presented in Table 2. Compared with non-Bt rice straw leachate at the same concentration, homozygous Bt rice straw leachate resulted in greater body length at 2 g L^−1^ but lower body length at 4 and 8 g L^−1^; body width was greater at 1 g L^−1^, whereas spine length was shorter at 8 g L^−1^. Compared with non-Bt rice straw leachate, heterozygous Bt rice straw leachate resulted in lower body length at 4 and 8 g L^−1^ and greater body width at 4 g L^−1^. No significant differences were observed in the remaining concentration-matched comparisons. Detailed statistical results are presented in Table S5. No significant differences in 21-day survival were detected between either Bt treatment and the corresponding non-Bt treatment (Table S4).

Reproductive parameters of first-generation *D. magna* are presented in Table 3. Compared with non-Bt rice straw leachate at the same concentration, homozygous Bt rice straw leachate resulted in a shorter time to first reproduction at 1 and 2 g L^−1^; first brood size was greater at 1 and 2 g L^−1^ but lower at 4 and 8 g L^−1^; the number of molts was lower at 1 and 4 g L^−1^; and total offspring production was greater at 1, 2, and 4 g L^−1^. Compared with non-Bt rice straw leachate, heterozygous Bt rice straw leachate resulted in a shorter time to first reproduction at 1, 2, and 8 g L^−1^; first brood size was greater at 1 g L^−1^ but lower at 4 g L^−1^; the number of molts was lower at 2 and 4 g L^−1^ but greater at 8 g L^−1^; and total offspring production was greater at all four concentrations (1, 2, 4, and 8 g L^−1^). No significant differences were observed in the remaining comparisons. Detailed statistical results are presented in Table S6.

#### 2.1.3. Effects on Catalase (CAT) Activity

**Figure 1:**
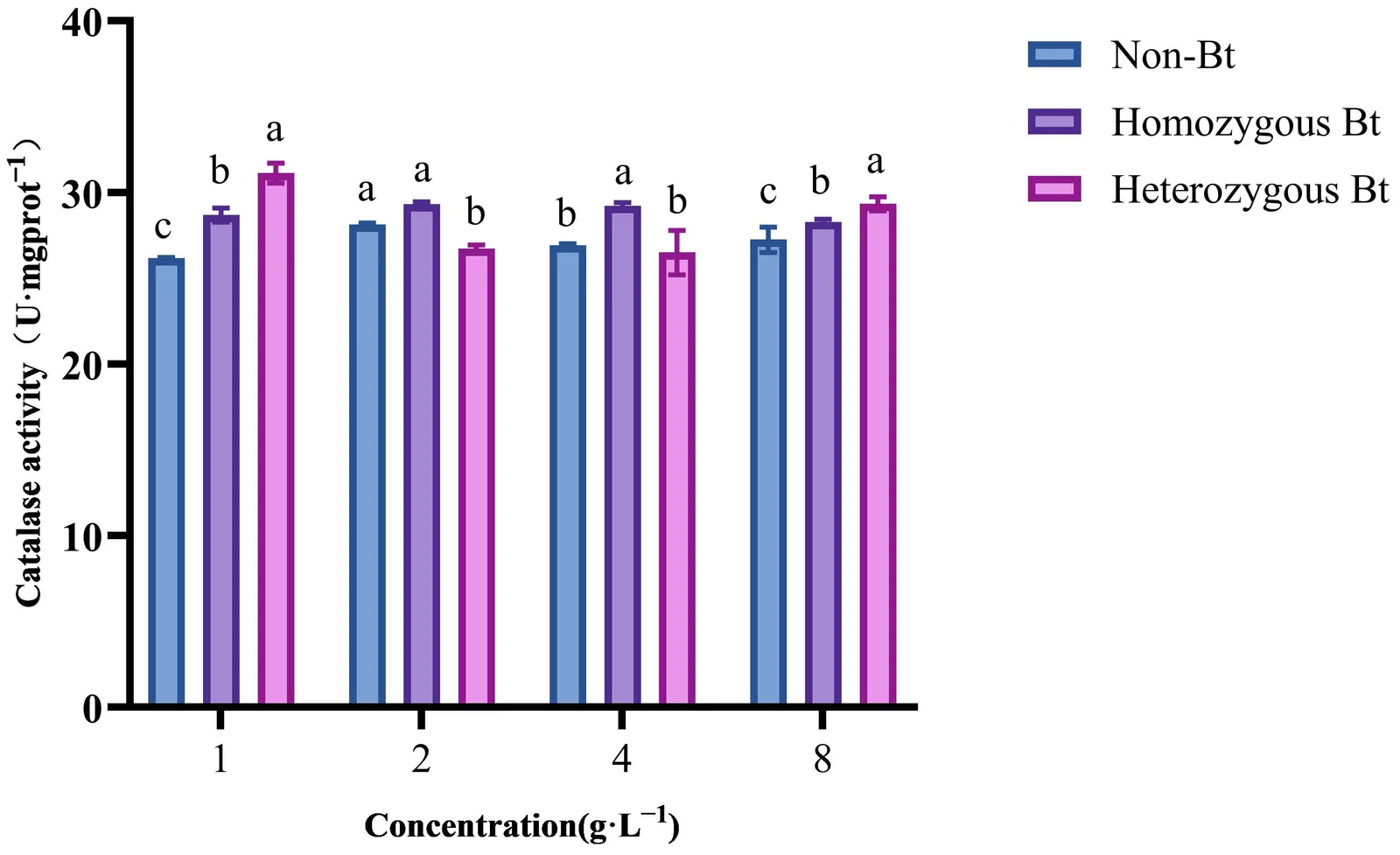
Effects of Bt rice straw leachates on catalase activity in first-generation *Daphnia magna* (means ± standard errors, *n* = 4; different lowercase letters at the same concentration indicate different multiple-comparison groupings, *p* < 0.05). Statistical results are presented in Tables S1 and S3a.

Catalase (CAT) activity in first-generation *D. magna* differed across straw leachate treatments (Figure 1). Compared with the concentration-matched non-Bt leachate, homozygous Bt leachate was originally reported to have no significant effect on CAT activity. Heterozygous Bt leachate was reported to significantly increase CAT activity by 18.91% and 7.63% at 1 and 8 g L^−1^, respectively, whereas no significant differences were reported at 2 or 4 g L^−1^. (Tables S1 and S3a).

#### 2.1.4. Effects on Peroxidase (POD) Activity

**Figure 2:**
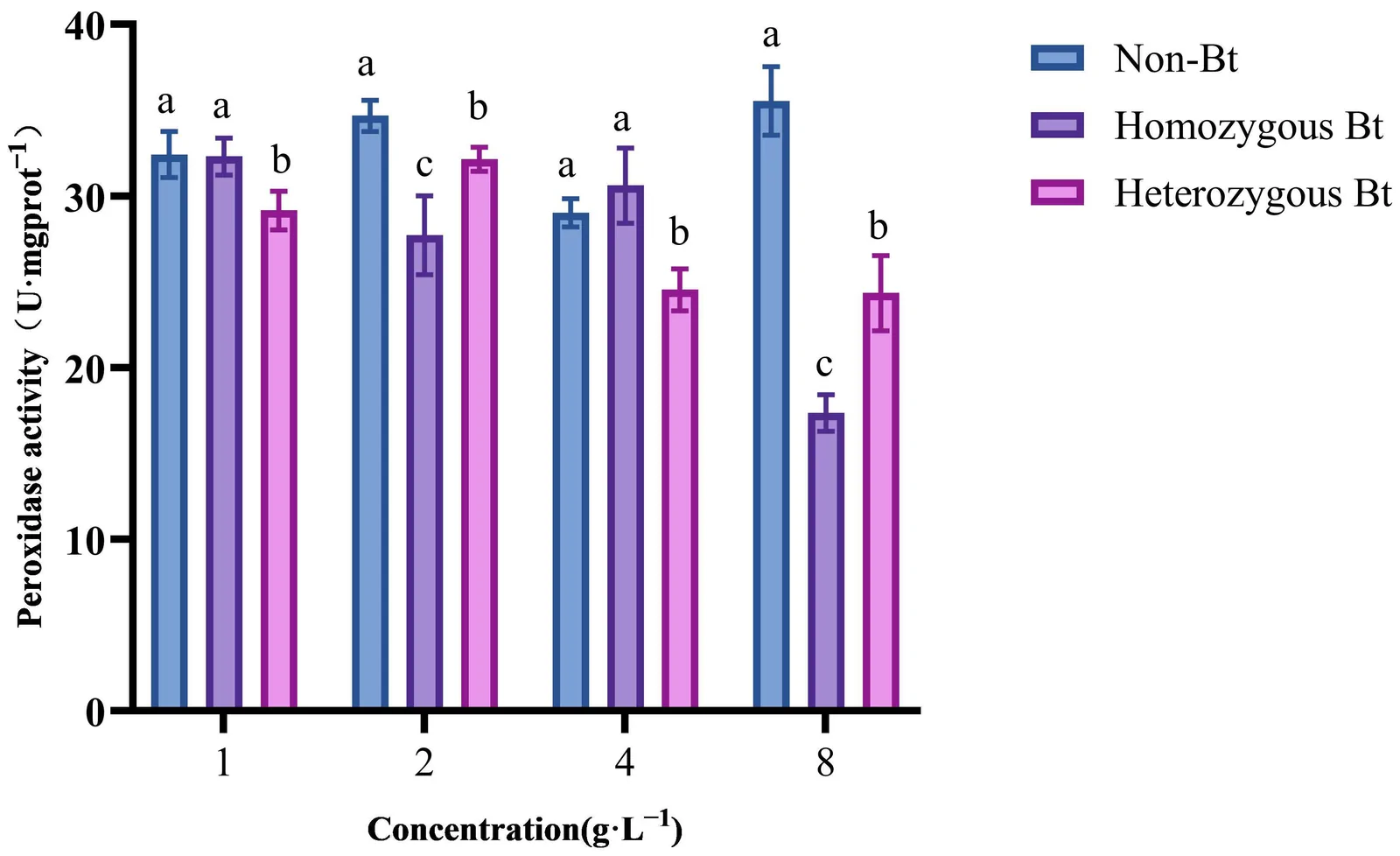
Effects of Bt rice straw leachates on peroxidase activity in first-generation *Daphnia magna* (means ± standard errors, *n* = 4; different lowercase letters at the same concentration indicate different multiple-comparison groupings, *p* < 0.05). Statistical results are presented in Tables S1 and S3b.

Rice straw leachate treatments produced different responses in first-generation Peroxidase (POD) activity (Figure 2). Compared with concentration-matched non-Bt leachate, homozygous Bt leachate was reported to increase POD activity by 5.4% at 4 g L^−1^ and decrease it by 25.11% and 52.47% at 2 and 8 g L^−1^, respectively; no significant difference was reported at 1 g L^−1^. Heterozygous Bt leachate was reported to decrease POD activity at 1, 2, 4, and 8 g L^−1^ by 11.21%, 7.87%, 18.26%, and 46.0%, respectively (Tables S1 and S3b).

#### 2.1.5. Effects on Superoxide Dismutase (SOD) Activity

**Figure 3:**
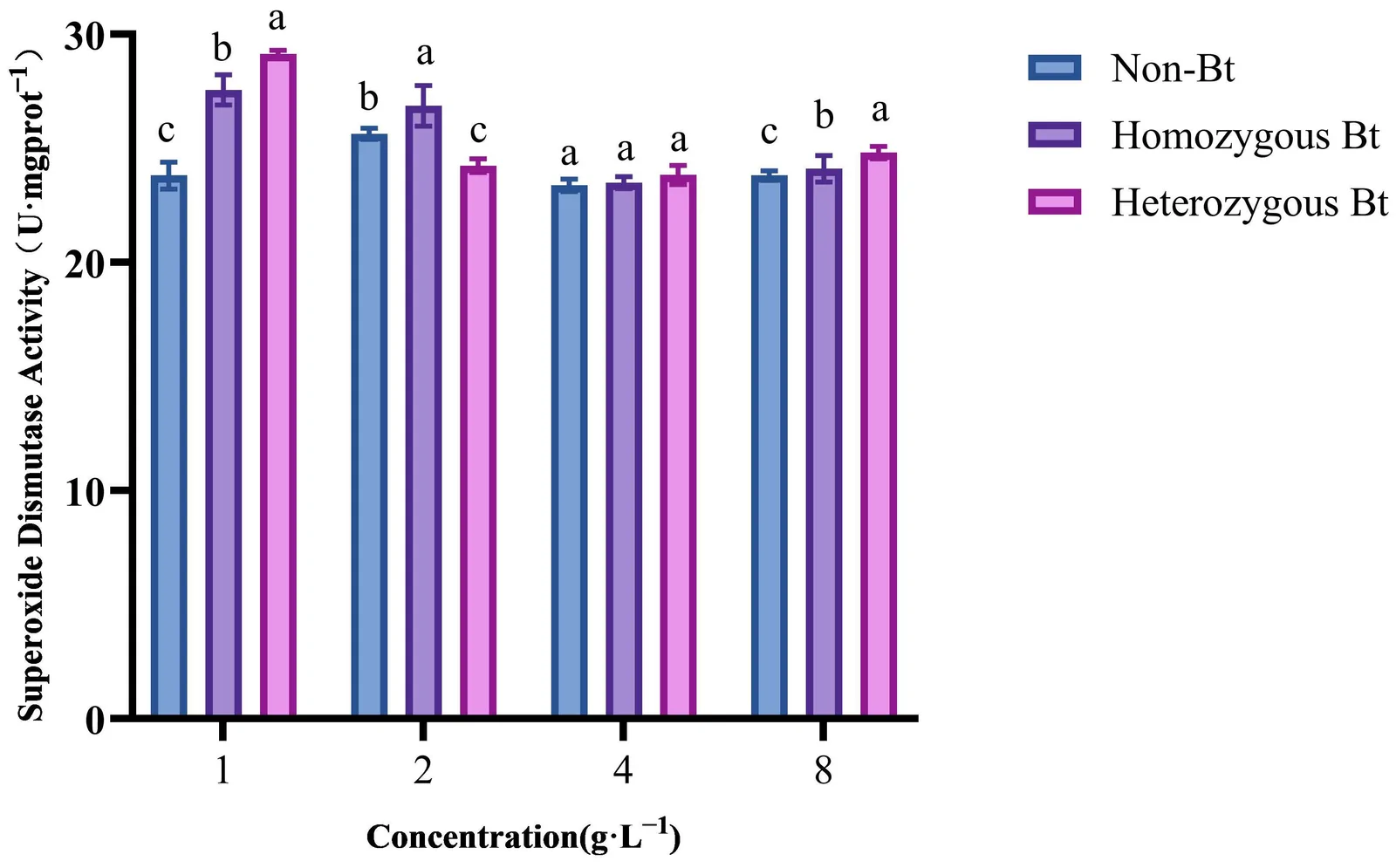
Effects of Bt rice straw leachates on superoxide dismutase activity in first-generation *Daphnia magna* (means ± standard errors, *n* = 4; different lowercase letters at the same concentration indicate different multiple-comparison groupings, *p* < 0.05). Statistical results are presented in Tables S1 and S3c.

The effects of rice straw leachates on first-generation Superoxide Dismutase (SOD) activity differed among treatments (Figure 3). Compared with concentration-matched non-Bt leachate, homozygous Bt leachate was originally reported to significantly increase SOD activity by 14.8% at 1 g L^−1^, with no significant differences at the other concentrations. Heterozygous Bt leachate was reported to significantly increase SOD activity by 20.69% and 7.4% at 1 and 8 g L^−1^, respectively, whereas no significant differences were reported at 2 or 4 g L^−1^ (Tables S1 and S3c).

#### 2.1.6. Effects on Malondialdehyde (MDA) Content

**Figure 4:**
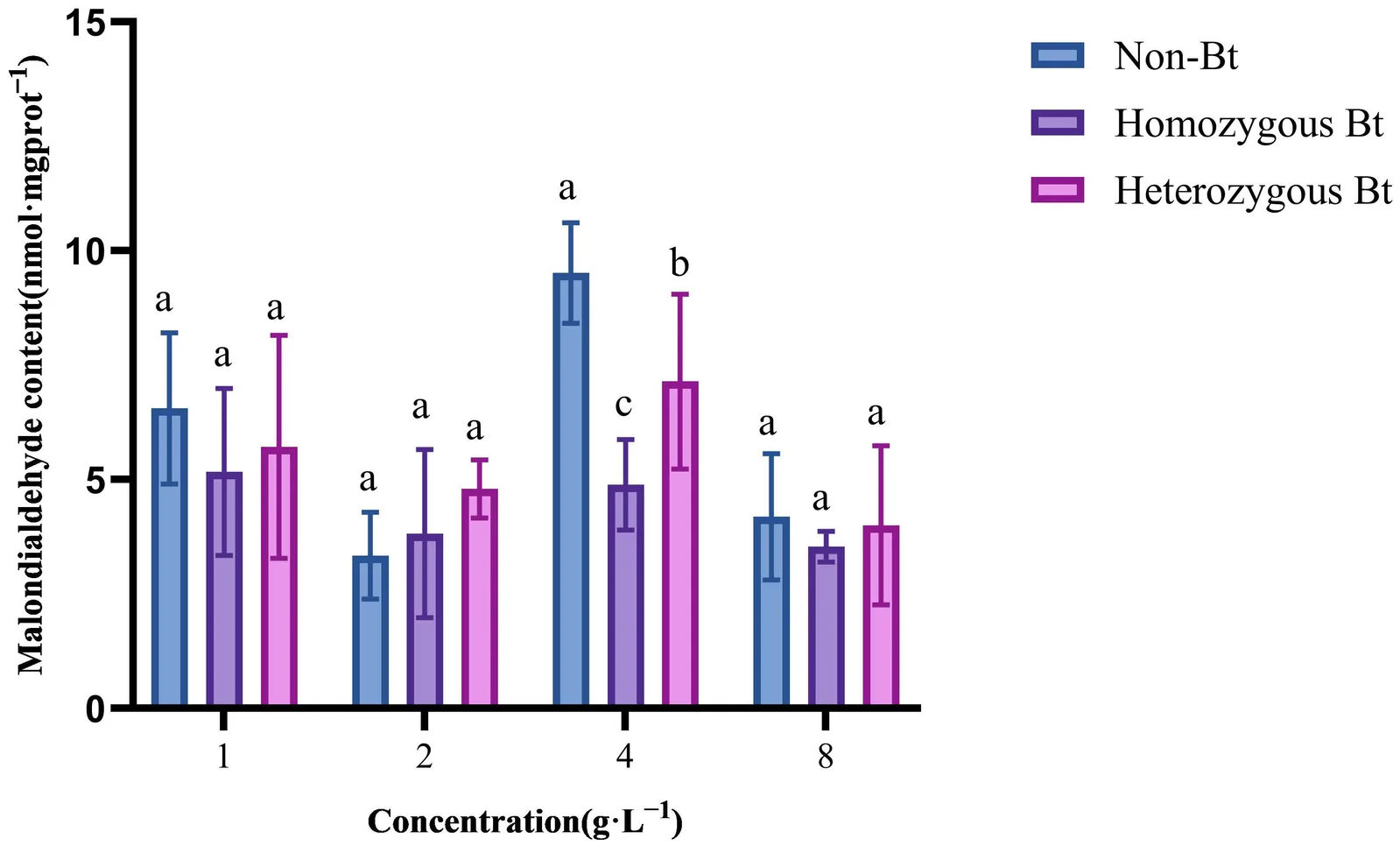
Effects of Bt rice straw leachates on malondialdehyde content in first-generation *Daphnia magna* (means ± standard errors, *n* = 4; different lowercase letters at the same concentration indicate different multiple-comparison groupings, *p* < 0.05). Statistical results are presented in Tables S1 and S3d.

Malondialdehyde (MDA) measurements in first-generation *D. magna* varied among straw leachate treatments (Figure 4). Compared with concentration-matched non-Bt leachate, homozygous Bt leachate was reported to significantly decrease MDA content by 48.9% at 4 g L^−1^, with no significant differences at other concentrations. Heterozygous Bt leachate was reported to significantly decrease MDA content by 26.7% at 4 g L^−1^, with no significant differences at the other concentrations (Tables S1 and S3d).

### 2.2. Effects of Bt Rice Straw Leachates on Second-Generation Daphnia magna

#### 2.2.1. Cry1Ab Concentrations in Second-Generation *D. magna*

Cry1Ab was detected in second-generation *D. magna* after 21 days of exposure (Table 4). The homozygous Bt treatment yielded 2.79, 3.82, 9.80, and 13.68 µg L^−1^ at nominal straw concentrations of 1, 2, 4, and 8 g L^−1^, respectively; the corresponding values under heterozygous Bt exposure were 0.87, 1.12, 1.28, and 1.88 µg L^−1^. Each genotype showed its highest measured value at 8 g L^−1^.

#### 2.2.2. Effects on Growth and Reproductive Parameters

Growth parameters of second-generation *D. magna* are presented in Table 5. Compared with non-Bt rice straw leachate at the same concentration, homozygous Bt rice straw leachate resulted in greater body length at 2 g L^−1^ but lower body length at 4 g L^−1^. Compared with non-Bt rice straw leachate, heterozygous Bt rice straw leachate resulted in lower body length at 4 g L^−1^ and lower body width at 2 g L^−1^. No significant differences were observed in the remaining concentration-matched comparisons. Detailed statistical results are presented in Table S7. Neither Bt treatment differed significantly from the corresponding non-Bt treatment in 21-day survival (Table S4).

Reproductive parameters of second-generation *D. magna* are presented in Table 6. Compared with non-Bt rice straw leachate at the same concentration, homozygous Bt rice straw leachate resulted in a shorter time to first reproduction at 1 g L^−1^ but a longer time at 4 g L^−1^; first brood size was greater at 1 g L^−1^ but lower at 4 g L^−1^; the number of molts was greater at 2 g L^−1^; and total offspring production was greater at 1, 2, 4, and 8 g L^−1^. Compared with non-Bt rice straw leachate, heterozygous Bt rice straw leachate resulted in a shorter time to first reproduction at 1 and 8 g L^−1^; first brood size was greater at 1 g L^−1^; the number of molts was greater at 8 g L^−1^; and total offspring production was greater at 1, 2, 4, and 8 g L^−1^. No significant differences were observed in the remaining comparisons. Detailed statistical results are presented in Table S8.

#### 2.2.3. Effects on Catalase (CAT) Activity

**Figure 5:**
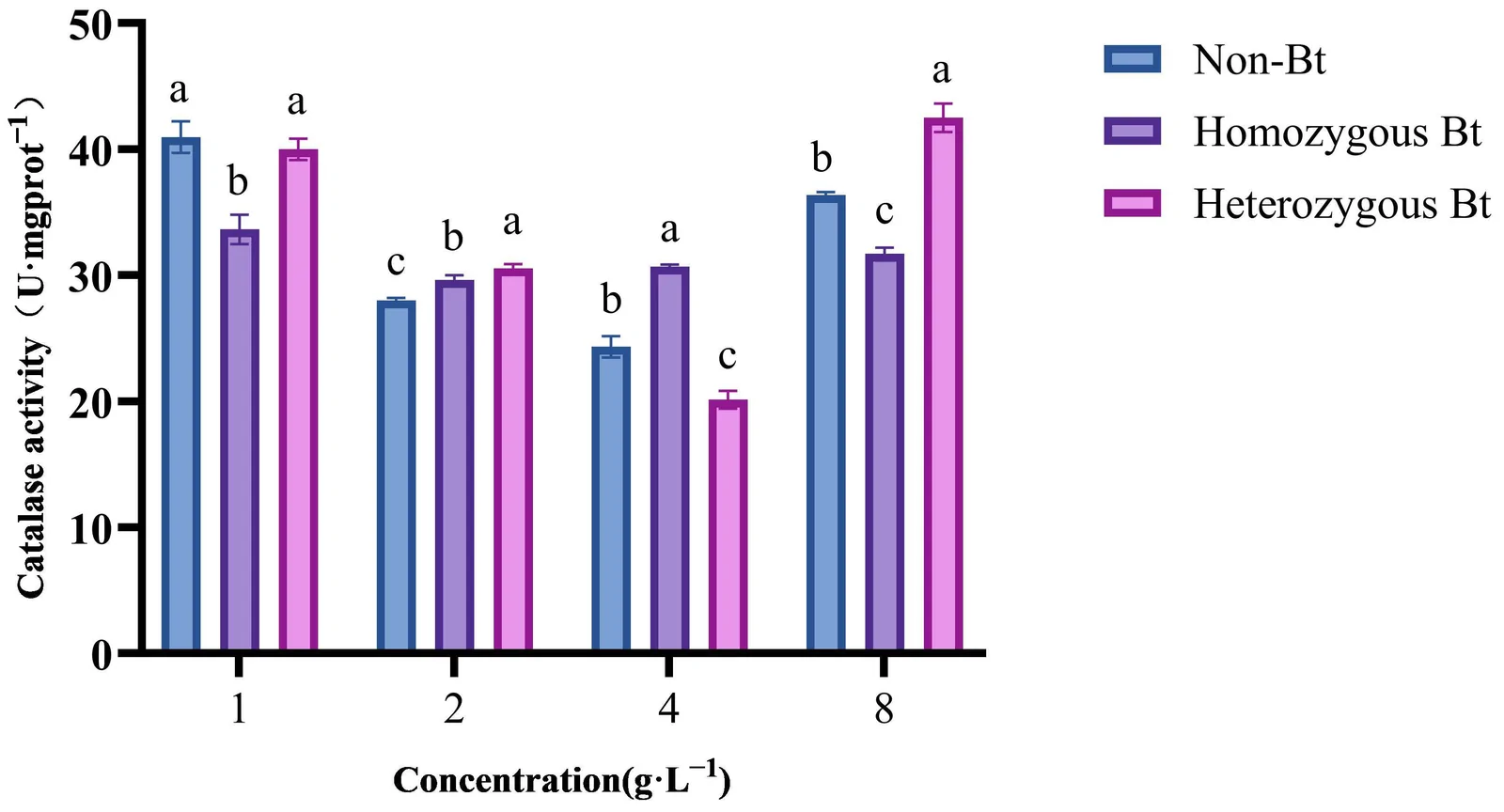
Effects of Bt rice straw leachates on catalase activity in second-generation *Daphnia magna* (means ± standard errors, *n* = 4; different lowercase letters at the same concentration indicate different multiple-comparison groupings, *p* < 0.05). Statistical results are presented in Tables S1 and S3e.

The effects of rice straw leachates on Catalase (CAT) activity in second-generation *D. magna* varied among treatments (Figure 5). Compared with concentration-matched non-Bt leachate, homozygous Bt leachate was reported to significantly increase CAT activity by 7.4% and 27.1% at 2 and 4 g L^−1^, respectively, with no significant differences at 1 or 8 g L^−1^. Heterozygous Bt leachate was reported to significantly increase CAT activity by 8.3% and 18.6% at 2 and 8 g L^−1^, respectively, and decrease it by 18.6% at 4 g L^−1^; no significant difference was reported at 1 g L^−1^ (Tables S1 and S3e).

#### 2.2.4. Effects on Peroxidase (POD) Activity

**Figure 6:**
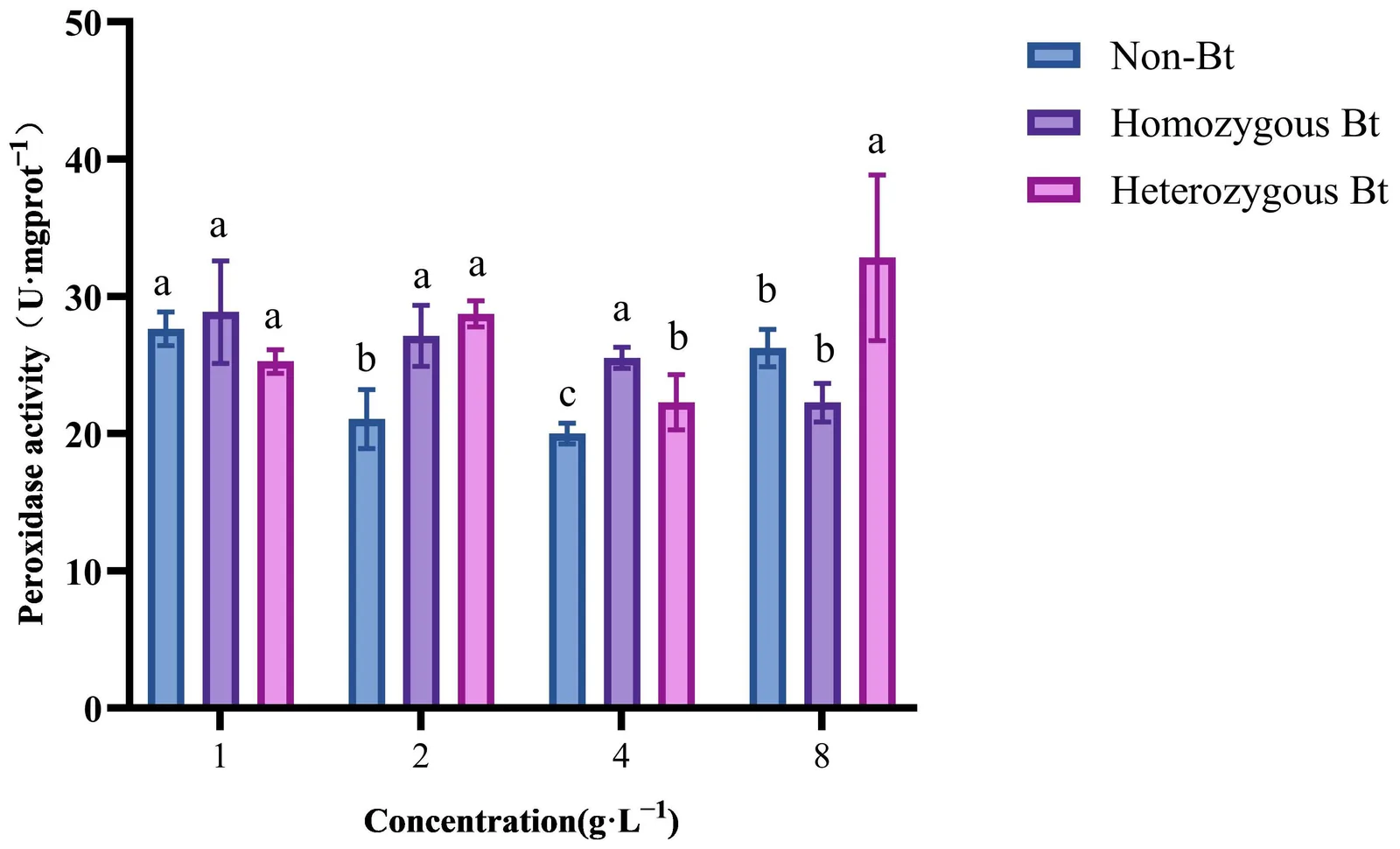
Effects of Bt rice straw leachates on peroxidase activity in second-generation *Daphnia magna* (means ± standard errors, *n* = 4; different lowercase letters at the same concentration indicate different multiple-comparison groupings, *p* < 0.05). Statistical results are presented in Tables S1 and S3f.

The effects of rice straw leachates on Peroxidase (POD) activity in second-generation *D. magna* varied among concentrations (Figure 6). Compared with concentration-matched non-Bt leachate, homozygous Bt leachate was reported to significantly increase POD activity by 26.2% and 27.5% at 2 and 4 g L^−1^, respectively, with no significant differences at 1 or 8 g L^−1^. Heterozygous Bt leachate was reported to significantly increase POD activity by 32.1% and 24.4% at 2 and 8 g L^−1^, respectively, with no significant differences at 1 or 4 g L^−1^ (Tables S1 and S3f).

#### 2.2.5. Effects on Superoxide Dismutase (SOD) Activity

**Figure 7:**
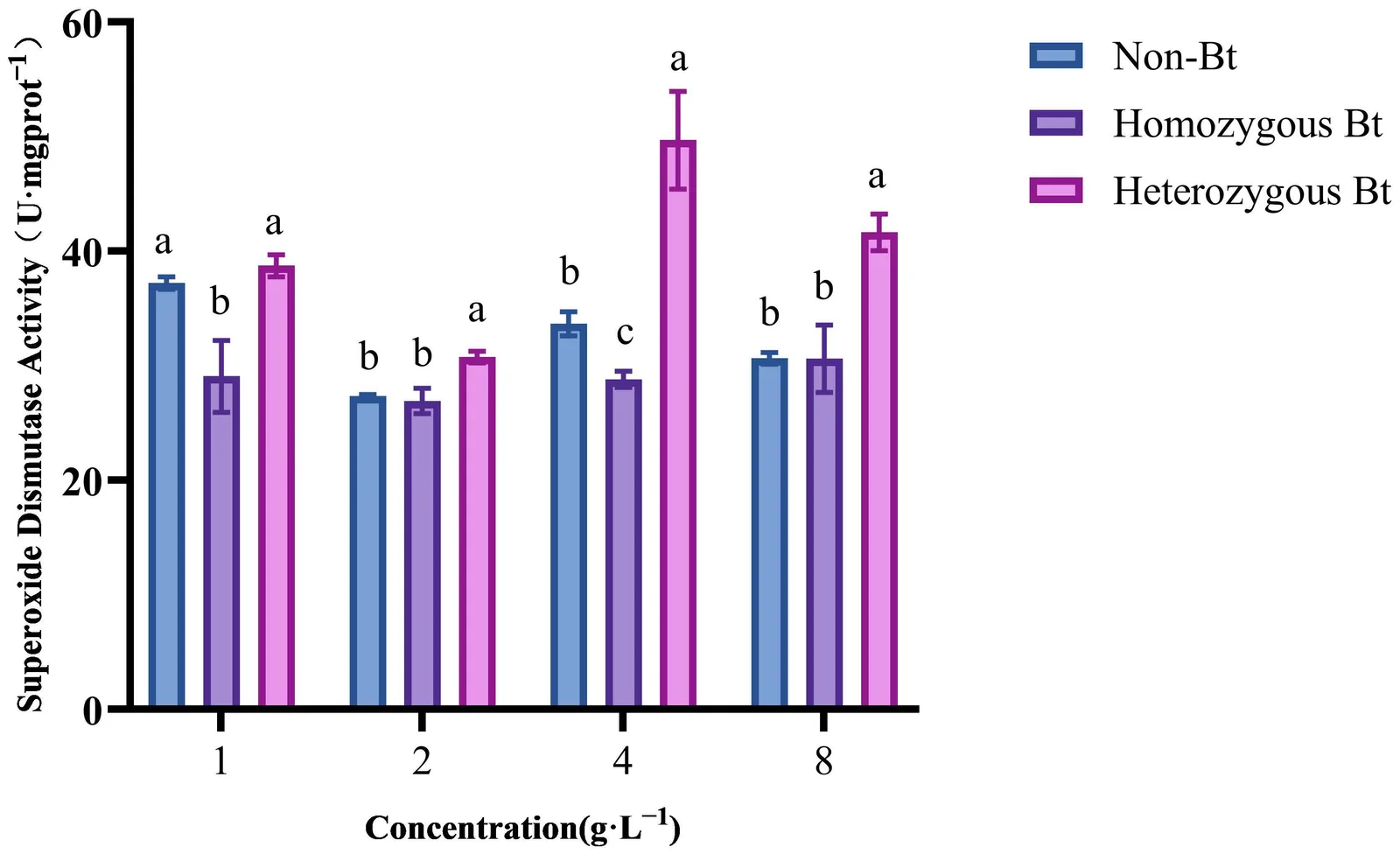
Effects of Bt rice straw leachates on superoxide dismutase activity in second-generation *Daphnia magna* (means ± standard errors, *n* = 4; different lowercase letters at the same concentration indicate different multiple-comparison groupings, *p* < 0.05). Statistical results are presented in Tables S1 and S3g.

Superoxide Dismutase (SOD) activity in second-generation *D. magna* varied among rice straw leachate treatments (Figure 7). Compared with concentration-matched non-Bt leachate, homozygous Bt leachate was reported to significantly decrease SOD activity by 21.6% and 14.7% at 1 and 4 g L^−1^, respectively, with no significant differences at 2 or 8 g L^−1^. Heterozygous Bt leachate was reported to significantly increase SOD activity by 9.1%, 41.2%, and 33.9% at 2, 4, and 8 g L^−1^, respectively, with no significant difference at 1 g L^−1^ (Tables S1 and S3g).

#### 2.2.6. Effects on Malondialdehyde (MDA) Content

**Figure 8:**
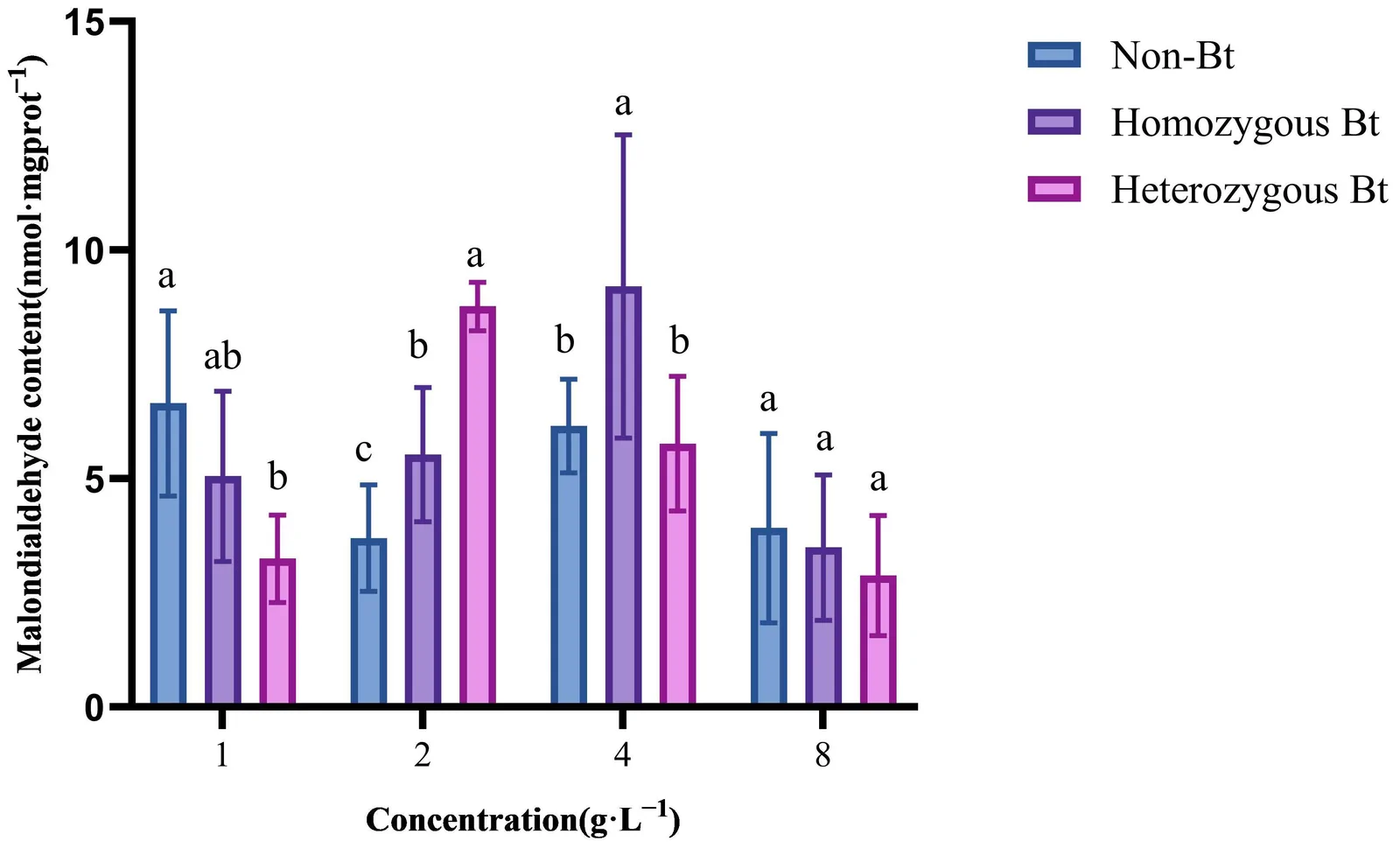
Effects of Bt rice straw leachates on malondialdehyde content in second-generation *Daphnia magna* (means ± standard errors, *n* = 4; different lowercase letters at the same concentration indicate different multiple-comparison groupings, *p* < 0.05). Statistical results are presented in Tables S1 and S3h.

Malondialdehyde (MDA)measurements in second-generation *D. magna* varied among treatments (Figure 8). Compared with concentration-matched non-Bt leachate, homozygous Bt leachate was reported to significantly increase MDA content by 55.3% and 51.7% at 2 and 4 g L^−1^, respectively, with no significant differences at the other concentrations. Heterozygous Bt leachate was reported to significantly increase MDA content by 138.2% at 2 g L^−1^, with no significant differences at the other concentrations (Tables S1 and S3h).

## 3. Discussion

After Bt rice straw is returned to paddy fields, straw-derived constituents and associated Cry proteins may enter paddy water; responses of non-target aquatic organisms therefore warrant assessment. *Daphnia magna* is sensitive to changes in aquatic conditions and has been used in related ecotoxicological research [10]. In this study, non-Bt straw leachate at the same nominal loading served as the comparator for homozygous and heterozygous Bt treatments across two generations. Some genotype-, concentration-, and endpoint-specific differences were observed, but their directions were not consistent. Across both generations, day 21 survival proportions did not differ significantly between Bt treatments and their concentration-matched non-Bt controls. The tested loadings of 1, 2, 4, and 8 g L^−1^ represent nominal straw mass concentrations rather than measured aqueous Cry1Ab concentrations; in the absence of continuous water-exposure and field evidence, these results cannot establish ecological risk under typical paddy-field conditions.

Cry1Ab was detected in *D. magna* samples at day 21, with generally higher values in homozygous than in corresponding heterozygous Bt treatments. However, a single endpoint measurement cannot represent aqueous Cry1Ab exposure throughout the 21-day period or establish continued bioaccumulation or rapid degradation in water. Griffiths et al. (2017) investigated transport or degradation of crop-derived Cry proteins, and studies by Chen et al. (2018, 2021) provide related aquatic non-target exposure context [21,22,23,24,25]. Differences in crop type, Cry protein, exposure medium, and sampling time must be considered when comparing studies. Concentration fluctuations may have occurred between medium renewals, but degradation kinetics from other studies cannot be assumed to have occurred here without corresponding monitoring data. In one archived three-day renewal observation, aqueous Cry1Ab measurements for homozygous Bt leachates at nominal straw concentrations of 1, 2, 4, and 8 g L^−1^ were 11.53, 20.35, 29.20, and 40.35 µg L^−1^ on day 1 and 3.41, 5.62, 10.61, and 13.52 µg L^−1^ on day 3, respectively. At the same loadings, the corresponding heterozygous Bt values were 2.53, 4.35, 5.20, and 7.35 µg L^−1^ on day 1 and 1.04, 1.62, 1.99, and 2.52 µg L^−1^ on day 3. These records suggest a decline in measured concentrations over this particular observation interval, not over every renewal cycle, and should not be directly compared with tissue-extract measurements obtained at day 21. The source and sampling batch of these archived water measurements require confirmation against the original laboratory records.

Under homozygous Bt rice straw leachate exposure, certain reproductive endpoints changed in the first generation at lower nominal concentrations, whereas selected growth parameters were lower at 8 g L^−1^; reduced Superoxide dismutase (SOD) activity and increased Malondialdehyde (MDA) measurements were observed at some concentrations in the second generation. These findings indicate endpoint-specific responses, but changes in enzyme activities or MDA alone do not demonstrate adaptive antioxidant stress, accumulated oxidative injury, or statistically established transgenerational toxicity. Although measured Cry1Ab concentrations differed between homozygous and heterozygous straw treatments, differences in straw-matrix constituents cannot be excluded, and genotype-dependent biochemical responses therefore cannot be attributed directly to Cry1Ab. The study by Wu et al. (2021) concerns a different crop-associated environmental context and cannot establish this causal mechanism [16]. Multigenerational studies by Nguyen et al. (2025) and Shim et al. (2025) support the rationale for longer-duration designs, but their stressors and statistical designs differ and do not demonstrate cumulative toxicity in the present experiment [19,20].

Under heterozygous Bt leachate exposure, certain reproductive and antioxidant-related endpoints changed in the first generation, and changes in MDA and antioxidant enzyme activities were observed at individual concentrations in the second generation. These responses neither establish stable antioxidant adaptation nor rule out long-term risk. Cry1Ab measurements tended to be lower in heterozygous than homozygous treatments, but other leachate constituents may also differ. A defensible interpretation is that whole-straw leachates from different rice genotypes elicited different endpoint responses; composition-matched controls and experiments supplementing purified Cry1Ab would be needed to isolate the protein’s contribution.

At the organismal level, the timing of first reproduction or offspring production changed at some lower straw loadings, whereas selected body dimensions or weights were reduced at higher loadings. Such concentration- and endpoint-specific responses were not consistently monotonic. Because a dedicated non-monotonic concentration–response model was not fitted, they should not be designated as low-dose hormesis. Studies of H23 transgenic rice, Bt maize, and Cry1C-expressing rice provide a comparative basis for examining non-target responses to different genetically modified crop materials [12,23,26,27]. However, differences in the plant material, Cry-protein type, exposure route, and measured endpoints mean that these studies do not jointly establish the safety of Cry proteins at every real-world exposure. The comparison by Chen et al. (2018) is particularly relevant because it evaluated leachates from Cry1C-expressing T1C-19 rice and its corresponding non-transgenic near-isoline MH63 over 21 days in *D. magna* [23]. Relative to leachate-free culture-medium controls, both straw leachates decreased certain survival, growth, or reproductive measures, whereas consistent significant differences between Bt and non-Bt straw types were generally not observed at matched leachate proportions. In the present Cry1Ab study, some reproductive and biochemical responses differed between rice types, but neither their direction nor their concentration dependence was uniform. These studies differ in Cry protein, straw source, leachate preparation, and control design; the findings therefore should not be equated or used to negate the importance of straw-matrix effects. The present study also detected differences in total phosphorus and total potassium among straw types (Table 7), which, in conjunction with evidence on genetic-background and matrix effects [7], prevents attributing all observed responses uniquely to Cry1Ab.

The physiological and biochemical endpoints—Catalase (CAT), Peroxidase (POD), and Superoxide dismutase (SOD) activities and Malondialdehyde (MDA)content—changed in different directions across concentrations and generations. For example, under first-generation homozygous Bt exposure, POD activity was reported to decrease by 25.11% and 52.47% at 2 and 8 g L^−1^, respectively, but to increase slightly by 5.4% at 4 g L^−1^. In the second generation, homozygous Bt treatments showed higher POD activity at 2 and 4 g L^−1^, while SOD was lower at some concentrations and MDA higher at others. These observations do not follow a single pattern in which greater loading always implies greater injury, and are more appropriately described as non-monotonic, endpoint-dependent patterns. Studies by Lv et al. (2022), Chen et al. (2018), and Eladel et al. (2019) involved other aquatic organisms or related biochemical responses and can inform comparisons among endpoints; they cannot collectively demonstrate that Bt straw suppresses POD in all settings [23,28,29]. Similarly, the algal study by Wang et al. (2014) and the study by Chen (2017) differ in organism, endpoint, or level of biological organization and cannot establish the absence of food-web effects in this experiment [11,30]. CAT, POD, SOD, and MDA are indirect redox-related markers; without independent reactive-oxygen or lipid-injury evidence and appropriate assay validation, these measurements cannot establish a mechanism of oxidative injury. The reported directional differences in POD provide descriptive examples only; a dedicated non-monotonic dose–response assessment was not performed. The significance of individual comparisons should also be reconciled with the original figures (Figure 2 and Figure 6) and the available statistical outputs.

Earlier feeding studies by Bøhn et al. (2008, 2010) reported reduced fitness in *D. magna* exposed to Bt maize diets, whereas Bøhn et al. (2016) tested purified Cry proteins; these should not be described as identical 42-day maize-feeding experiments [31,32,33]. Cuhra et al. (2015) compared soybean diets from different production systems rather than Cry1Ab-specific exposures [34], and Holderbaum et al. (2015) studied dietary exposure to genetically modified maize materials rather than rice straw leachates [35]. Differences from the present findings may relate to food quality, crop background, Cry-protein type, duration, or exposure route, but none of these explanations was experimentally isolated in this study. Guidance by EFSA (2010) and non-target risk-assessment criteria discussed by Romeis et al. (2013) emphasize suitable comparators, exposure characterization, and evidence quality [36,37]; they do not justify dismissing earlier studies reporting adverse responses as methodologically defective. More specifically, Bøhn et al. (2008) More specifically, higher mortality, a lower proportion of females reaching maturity, and reduced total egg production were recorded in *D. magna* fed Cry1Ab‑transgenic maize meal compared with organisms receiving the matching non‑transgenic control material [32]. Subsequent life‑table and population‑parameter assessments reinforced these observations [33]. Separate full life‑cycle exposures to purified Cry1Ab, Cry2Aa, and their mixture detected negative effects on survival, growth and reproduction at several higher protein concentrations [34].These findings merit consideration; however, dietary intake and mg L^−1^-scale purified-protein exposure are not directly equivalent to the straw-leachate system studied here. The absence of a significant day 21 survival difference in the present experiment does not contradict results obtained under different exposure conditions.

This controlled laboratory study describes growth, reproductive, and biochemical responses of two generations of *D. magna* to different rice straw leachates, but its interpretation is limited by several factors. First, aqueous Cry1Ab concentrations and key water-quality parameters—such as pH, dissolved oxygen, ammonia nitrogen, and electrical conductivity—were not continuously recorded throughout exposure, limiting separation of protein exposure from water-chemistry effects. Second, no joint generation × rice genotype × concentration interaction analysis was performed; cumulative toxicity or adaptive responses across generations therefore cannot be established. This experiment did not extend to population- or community-level endpoints [3]. Future work should use more field-relevant loadings and exposure scenarios, include non-Bt comparator matrices of matched composition and purified Cry1Ab-supplementation treatments, and monitor Cry1Ab and water quality throughout exposure. Transgenerational effects should be tested with verified independent sampling units. Quantitative PCR or transcriptomic methods could then help formulate and test hypotheses about oxidative stress and nutritional metabolism [38], but molecular findings would need to be interpreted alongside physiological endpoints.

## 4. Materials and Methods

### 4.1. Experimental Materials

*Daphnia magna*: The test animals were obtained from a monoclonal laboratory population maintained over several generations in fully aerated tap water. Culture conditions were 25 °C, approximately 3000 lx, and a 14 h light: 10 h dark photoperiod; water was renewed three times per week. The animals were fed *Scenedesmus obliquus*. Actively swimming neonates aged 6–24 h were selected for experimental exposure to minimize initial differences in developmental stage. The selection of young neonates and the 21‑day reproductive observation period followed the general design principles of the OECD *Daphnia* reproduction test guideline [39,40,41].

*Scenedesmus obliquus*: The algal strain was obtained from the Freshwater Algae Culture Collection of the Institute of Hydrobiology, Chinese Academy of Sciences (Beijing, China). One week before exposure, 100 mL of BG-11 medium was dispensed aseptically into a sterile 1000 mL Erlenmeyer flask, inoculated with *S. obliquus*, mixed, and incubated in a light-controlled growth chamber at 24 ± 1 °C and 4000 lx with a 12 h light:12 h dark photoperiod. The flask was swirled three to five times per day and supplemented with 100 mL of fresh BG-11 medium every 24 h. Algal cells in exponential growth were used as Daphnia feed.

Rice straw: The homozygous Bt rice line T775 expressing Cry1Ab, the heterozygous Bt rice (T775 F_1_ hybrid) and the non-Bt variety ‘Tianyou Huazhan’ were all supplied by the Rice Research Institute of the Guangdong Academy of Agricultural Sciences (Guangzhou, China). Rice straw (stem and leaf tissue) was harvested at the grain maturity stage. Fresh straw was placed in a cool, well-ventilated indoor environment and air-dried at room temperature until constant weight was achieved. After air-drying, the straw was chopped using a plant grinder, sieved, and fragments shorter than 3 cm were retained. The chopped straw was thoroughly mixed to minimise sampling heterogeneity. The material was sealed in plastic bags, stored at −20 °C away from light, and set aside for use in the preparation of the extract [42]. An extract from non-GM ‘Tianyou Huazhan’ rice straw was used as a concentration-matched control treatment.

#### 4.1.1. Preparation of Rice Straw Leachates

For each rice genotype, 8.0 g of chopped rice straw was added to a 1 L Erlenmeyer flask containing 1000 mL of BG-11 medium. The flask was sealed and incubated on a shaker at 100 rpm and 25 °C for 24 h. The suspension was centrifuged at 4000 rpm for 10 min at room temperature, and the supernatant was collected as the stock leachate. Based on the initial straw mass divided by the extraction volume, the nominal concentration of the stock leachate was 8 g L^−1^. The stock leachate was diluted with the experimental exposure medium to prepare working leachates at nominal straw-loading concentrations of 1, 2, and 4 g L^−1^; the undiluted stock leachate was used directly for the 8 g L^−1^ treatment. In this study, leachate concentrations were expressed as straw mass concentrations (g L^−1^). The physicochemical properties of the stock leachates from the different rice types are presented in Table 7.

#### 4.1.2. Experimental Design

The design comprised three straw types (non-Bt, homozygous Bt, and heterozygous Bt) and four nominal loading concentrations (1, 2, 4, and 8 g L^−1^). For each of the 12 treatment combinations, 40 separate 50 mL plastic exposure cups were prepared, each containing 20 mL of leachate and one 6–24 h-old *D. magna* neonate. The individual animal and its cup were the initial units for survival, growth, and reproduction monitoring. Exposure lasted 21 days per generation at 25 °C, 3000 lx, and a 14 h light:10 h dark photoperiod. Exposure media were replaced completely every three days, and animals were fed *S. obliquus* daily. Concentrations represented a controlled eightfold range of nominal laboratory straw loading rather than measured field-realistic aqueous Cry1Ab levels.

In the first generation, individual survival, molting, age at first reproduction, first-brood offspring, and total offspring over 21 days were recorded daily. Surviving animals were measured for body length, body width, spine length, and weight at day 21, and samples intended for Cry1Ab and biochemical assays were stored at −80 °C. Because one *D. magna* provided insufficient biomass, four non-overlapping pooled samples were assembled from surviving individuals per treatment. Each pool was independently homogenized, centrifuged, and assayed. The independent pooled homogenate was treated as the biological replicate for Catalase (CAT), Peroxidase (POD), Superoxide dismutase (SOD), and Malondialdehyde (MDA) (*n* = 4 per treatment); neither animals within pools nor repeat readings from one homogenate were counted again as independent replicates. During the recorded three-day renewal cycle, 500 µL exposure medium was sampled daily and preserved for aqueous Cry1Ab measurement.

Second-generation neonates aged 6–24 h were obtained from the second or third brood of first-generation mothers in the matching treatment. The recorded design specifies one neonate per distinct maternal donor, with a target of 40 independently sourced second-generation individuals per treatment. The second generation used the same nominal concentrations, cup exposures, culture conditions, feeding, medium renewal, and day 21 measurements. Maternal identifiers and the exact timing of selection relative to first-generation mortality should be checked against the individual rearing log to verify that all 40 donors were distinct for every treatment. Each generation was analysed separately.

#### 4.1.3. Determination of Cry1Ab Protein Concentrations

Using the Agdia Bt‑Cry1Ab assay kit (Agdia, Inc., Elkhart, IN, USA), the Cry1Ab protein content in rice straw extracts and *Daphnia magna* samples was determined via an enzyme-linked immunosorbent assay (ELISA). The testing procedure followed previously reported studies on Bt protein exposure in *Daphnia magna* [22,23,43]. Extract samples: The experimental solution was replaced every 3 days; during this period, 500 μL of the exposure medium was collected daily and stored for analysis. Samples were mixed thoroughly prior to testing and diluted with phosphate-buffered saline containing Tween (PBST) as needed. Biological samples: Collect *Daphnia magna* from each generation after 21 days of exposure and store at −80 °C. Add PBST extraction buffer to the frozen samples and extract by intermittent shaking for 30 min; centrifuge at 4 °C and 12,000 rpm for 10 min, then collect the supernatant and dilute as needed. Prepare six Cry1Ab standard solutions in a gradient. Incubate the standards and test samples (100 μL per well) for 2 h, then wash five times with PBST; add 100 μL of enzyme conjugate to each well, continue incubation for 2 h, and wash with PBST 5 more times; and add 100 μL of TMB substrate, incubate in the dark for 20 min, and measure the absorbance at a wavelength of 650 nm. Calculate the Cry1Ab concentration based on the standard curve and correct it for the sample dilution factor.

#### 4.1.4. Measurement of Growth and Reproductive Parameters

Survival, molting, age at first reproduction, first-brood offspring number, and total offspring produced during the 21-day period were recorded individually. At day 21, surviving animals were weighed and their body length, body width, and spine length were measured under a stereomicroscope.

#### 4.1.5. Determination of Antioxidant Enzyme Activities and Malondialdehyde (MDA)

*Daphnia magna* samples were weighed and homogenized mechanically in an ice-water bath with physiological saline at a sample mass-to-volume ratio of 1:9 (g:mL), yielding a 10% homogenate. After centrifugation at 3000 rpm for 10 min, the supernatant was used to measure Catalase (CAT), Peroxidase (POD), and Superoxide dismutase (SOD) activities, with reference to Gao et al. (2018) and [44]. SOD activity was assessed by inhibition of nitroblue tetrazolium (NBT) photoreduction, CAT activity by hydrogen peroxide decomposition, and POD activity by guaiacol oxidation in the presence of hydrogen peroxide. CAT, POD, and SOD were evaluated as antioxidant-related enzyme activities; Malondialdehyde (MDA), a lipid-peroxidation biomarker, was quantified using the thiobarbituric-acid (TBA) colorimetric method with commercially available assay kits (Nanjing Jiancheng Bioengineering Institute, Nanjing, China). Absorbance values were recorded at 450 nm, 532 nm, and 600 nm to correct for sugar interference and sample turbidity. Calculations followed the manufacturer-provided formula based on the molar extinction coefficient of 155 L·mmol^−1^·cm^−1^ for the MDA-TBA adduct; no external MDA standard calibration curve was constructed. All MDA results were normalized to the total soluble protein content of each homogenate and expressed as nmol·mgprot^−1^.

### 4.2. Statistical Analysis

Data were compiled using Microsoft Office, and figures were prepared using GraphPad Prism11.0. Statistical analyses were conducted using SPSS 17.0 and supplementary calculations. Results are presented as means ± standard errors (SE), and the two generations were analyzed separately. Additional analyses of growth and reproductive endpoints were based on the reported summary statistics. Two-way analysis of variance was used to evaluate rice type, nominal leachate concentration, and their interaction; Bt treatments were compared with concentration-matched non-Bt controls using two-sided Welch *t*-tests, with Holm adjustment for multiple comparisons [45,46]. The original Duncan multiple range test groupings were retained in the main-text tables [47,48]. Catalase (CAT), Peroxidase (POD), Superoxide dismutase (SOD), and Malondialdehyde (MDA) were analyzed using separate two-way analyses of variance for each generation, with four independently prepared pooled homogenates per treatment. Model assumptions were assessed using the Shapiro–Wilk and Brown–Forsythe tests [49,50], and sensitivity analyses were conducted. Day 21 survival was compared using two-sided Fisher exact tests [51]. Statistical significance was defined as *p* < 0.05. Detailed results are provided in Supplementary Tables S1–S9.

## 5. Conclusions

Straw leachates from non-Bt Tianyou Huazhan, homozygous Bt T775, and heterozygous Bt T775 F_1_ rice were tested in *D. magna* at nominal straw concentrations of 1, 2, 4, and 8 g L^−1^ for 21 days in each of two generations. Survival, growth, reproduction, and Catalase (CAT), Peroxidase (POD), Superoxide dismutase (SOD), and Malondialdehyde (MDA) responses were evaluated. Mean responses differed in direction for certain endpoints across rice types and loadings, without a uniform concentration-dependent pattern. Concentration-matched comparisons of day 21 survivor counts detected no significant differences between Bt and non-Bt groups. The antioxidant and MDA findings alone do not establish oxidative injury, and descriptive differences between generations do not demonstrate cumulative transgenerational toxicity. Further field or semi-field validation and long-term population-level studies are needed under environmentally relevant exposure conditions, with longitudinal Cry1Ab and water-quality measurements and compositionally comparable control materials.

## Figures and Tables

**Table 1:** Cry1Ab content in first-generation *Daphnia magna* exposed to Bt rice straw leachates (µg L^−1^). Note: Values are means ± SE; different lowercase letters indicate Duncan groupings among concentrations within the same rice type (*p* < 0.05).

Treatment	1 g·L^−1^	2 g·L^−1^	4 g·L^−1^	8 g·L^−1^
Homozygous Bt	2.42 ± 0.23 c	4.35 ± 0.24 c	9.29 ± 0.13 b	15.35 ± 0.51 a
Heterozygous Bt	0.93 ± 0.43 bc	1.08 ± 0.11 bc	1.2 ± 0.32 b	1.75 ± 0.44 a

**Table 2:** Effects of Bt rice straw leachates on growth parameters of first-generation *Daphnia magna.* Note: Values represent means ± standard error of the mean (SEM). Different lowercase letters within the same column indicate significant differences among treatments under the same nominal straw-loading concentration (Duncan’s multiple-range test, *p* < 0.05). *n* denotes the number of surviving animals on day 21 out of 40 initially exposed individuals per treatment. Between-treatment *p* values are presented in Table S5, and survival statistics in Table S4.

Treatment	Conc. (g·L^−1^)	Body Length (mm)	Body Width (mm)	Spine Length (mm)	Body Weight (mg)	Survival Rate (%, *n*)
Non-Bt	—	2.52 ± 0.03 a	1.63 ± 0.02 a	0.41 ± 0.01 b	2.49 ± 0.06 a	100 (40)
Homozygous Bt	1	2.65 ± 0.04 a	1.76 ± 0.03 a	0.36 ± 0.08 b	2.73 ± 0.08 a	100 (40)
Heterozygous Bt	1	2.48 ± 0.04 ab	1.69 ± 0.06 ab	0.45 ± 0.03 b	2.54 ± 0.08 a	100 (40)
Non-Bt	2	2.61 ± 0.02 a	1.76 ± 0.03 a	0.52 ± 0.02 ab	2.35 ± 0.09 a	100 (40)
Homozygous Bt	2	2.73 ± 0.03 a	1.82 ± 0.04 a	0.57 ± 0.04 a	2.36 ± 0.21 a	95 (38)
Heterozygous Bt	2	2.62 ± 0.07 a	1.72 ± 0.01 a	0.58 ± 0.05 a	2.67 ± 0.12 ab	97.5 (39)
Non-Bt	4	2.72 ± 0.05 a	1.66 ± 0.04 a	0.63 ± 0.03 a	2.45 ± 0.05 a	95 (38)
Homozygous Bt	4	2.41 ± 0.02 ab	1.76 ± 0.02 a	0.55 ± 0.03 a	2.16 ± 0.12 ab	92.5 (37)
Heterozygous Bt	4	2.51 ± 0.03 a	1.83 ± 0.03 a	0.61 ± 0.07 a	2.43 ± 0.11 ab	92.5 (37)
Non-Bt	8	2.56 ± 0.02 a	1.64 ± 0.01 a	0.57 ± 0.03 a	1.94 ± 0.08 b	90 (36)
Homozygous Bt	8	2.28 ± 0.04 b	1.69 ± 0.04 ab	0.44 ± 0.01 b	1.84 ± 0.07 b	87.5 (35)
Heterozygous Bt	8	2.42 ± 0.03 ab	1.74 ± 0.04 a	0.53 ± 0.09 ab	2.20 ± 0.02 c	90 (36)

**Table 3:** Effects of Bt rice straw leachates on reproductive parameters of first-generation *Daphnia magna.* Note: Values represent means ± standard error of the mean (SEM). Different lowercase letters within the same column indicate significant differences among treatments under the same nominal straw-loading concentration (Duncan’s multiple-range test, *p* < 0.05). Between-treatment *p* values are presented in Table S6.

Treatment	Conc. (g·L^−1^)	Time to First Reproduction (d)	First Brood Size	Number of Molts	Total Offspring
Non-Bt	—	9.32 ± 0.12 a	6.35 ± 0.20 b	8.33 ± 0.12 a	28.31 ± 0.58 b
Homozygous Bt	1	8.32 ± 0.21 ab	8.34 ± 0.25 a	7.43 ± 0.20 ab	36.25 ± 0.57 ab
Heterozygous Bt	1	8.14 ± 0.32 ab	8.21 ± 0.05 a	8.58 ± 0.11 a	38.67 ± 0.55 ab
Non-Bt	2	8.92 ± 0.11 ab	7.34 ± 0.25 a	8.43 ± 0.21 a	37.20 ± 0.75 a
Homozygous Bt	2	7.81 ± 0.25 b	8.21 ± 0.18 a	8.15 ± 0.12 a	49.18 ± 0.39 a
Heterozygous Bt	2	7.68 ± 0.11 c	7.58 ± 0.24 a	7.33 ± 0.25 ab	43.36 ± 1.02 a
Non-Bt	4	8.52 ± 0.12 ab	8.41 ± 0.19 a	9.42 ± 0.25 a	25.22 ± 1.05 b
Homozygous Bt	4	8.36 ± 0.13 ab	7.37 ± 0.12 a	8.28 ± 0.23 a	33.51 ± 0.52 ab
Heterozygous Bt	4	8.06 ± 0.15 ab	6.89 ± 0.35 ab	8.37 ± 0.24 a	33.28 ± 0.27 b
Non-Bt	8	10.12 ± 0.14 a	7.65 ± 0.15 a	7.68 ± 0.29 a	24.95 ± 1.24 b
Homozygous Bt	8	9.51 ± 0.23 b	7.03 ± 0.03 ab	8.13 ± 0.21 a	25.34 ± 0.65 b
Heterozygous Bt	8	8.21 ± 0.26 ab	7.33 ± 0.26 a	8.74 ± 0.15 a	29.37 ± 0.86 b

**Table 4:** Cry1Ab content in second-generation *Daphnia magna* exposed to Bt rice straw leachates (µg L^−1^). Note: Values are means ± SE; different lowercase letters within the same rice type indicate Duncan multiple-comparison groupings among concentrations (*p* < 0.05).

Treatment	1 g·L^−1^	2 g·L^−1^	4 g·L^−1^	8 g·L^−1^
Homozygous Bt	2.79 ± 0.12 c	3.82 ± 0.14 c	9.8 ± 0.19 b	13.68 ± 0.75 a
Heterozygous Bt	0.87 ± 0.32 c	1.12 ± 0.25 b	1.28 ± 0.15 b	1.88 ± 0.17 a

**Table 5:** Effects of Bt rice straw leachates on growth parameters of second-generation *Daphnia magna*. Note: Values represent means ± standard error of the mean (SEM). Different lowercase letters within the same column indicate significant differences among treatments under the same nominal straw-loading concentration (Duncan’s multiple-range test, *p* < 0.05). *n* denotes the number of surviving animals on day 21 out of 40 initially exposed individuals per treatment. Between-treatment *p* values are presented in Table S7, and survival statistics in Table S4.

Treatment	Conc. (g·L^−1^)	Body Length (mm)	Body Width (mm)	Spine Length (mm)	Body Weight (mg)	Survival Rate (%, *n*)
Non-Bt	—	2.53 ± 0.03 a	1.63 ± 0.02 a	0.35 ± 0.01 ab	2.39 ± 0.06 a	100 (40)
Homozygous Bt	1	2.65 ± 0.04 a	1.71 ± 0.03 a	0.43 ± 0.08 a	2.53 ± 0.08 a	97.5 (39)
Heterozygous Bt	1	2.48 ± 0.04 a	1.69 ± 0.06 a	0.39 ± 0.03 b	2.42 ± 0.08 a	100 (40)
Non-Bt	2	2.71 ± 0.02 a	1.86 ± 0.03 a	0.47 ± 0.02 a	2.35 ± 0.09 a	100 (40)
Homozygous Bt	2	2.83 ± 0.03 a	1.82 ± 0.04 a	0.55 ± 0.04 a	2.44 ± 0.21 a	95 (38)
Heterozygous Bt	2	2.72 ± 0.07 a	1.72 ± 0.01 a	0.52 ± 0.05 a	2.57 ± 0.12 a	97.5 (39)
Non-Bt	4	2.82 ± 0.05 a	1.71 ± 0.04 a	0.53 ± 0.03 a	2.45 ± 0.05 a	97.5 (39)
Homozygous Bt	4	2.51 ± 0.02 a	1.75 ± 0.02 a	0.51 ± 0.03 a	2.16 ± 0.12 ab	92.5 (37)
Heterozygous Bt	4	2.61 ± 0.03 a	1.73 ± 0.03 a	0.58 ± 0.07 a	2.41 ± 0.11 a	95 (38)
Non-Bt	8	2.46 ± 0.02 a	1.67 ± 0.01 a	0.42 ± 0.03 a	2.21 ± 0.08 a	92.5 (37)
Homozygous Bt	8	2.43 ± 0.04 a	1.69 ± 0.04 ab	0.45 ± 0.01 a	1.94 ± 0.07 b	90 (36)
Heterozygous Bt	8	2.52 ± 0.03 a	1.64 ± 0.04 ab	0.51 ± 0.09 a	2.31 ± 0.02 a	90 (36)

**Table 6:** Effects of Bt rice straw leachates on reproductive parameters of second-generation *Daphnia magna.* Note: Values represent means ± standard error of the mean (SEM). Different lowercase letters within the same column indicate significant differences among treatments under the same nominal straw-loading concentration (Duncan’s multiple-range test, *p* < 0.05). Between-treatment *p* values are presented in Table S8.

Treatment	Conc. (g·L^−1^)	Time to First Reproduction (d)	First Brood Size	Number of Molts	Total Offspring
Non-Bt	—	9.28 ± 0.11 a	6.56 ± 0.21 b	7.35 ± 0.21 ab	29.51 ± 0.48 b
Homozygous Bt	1	8.29 ± 0.24 ab	8.25 ± 0.24 a	7.78 ± 0.20 a	33.65 ± 0.32 ab
Heterozygous Bt	1	8.54 ± 0.14 ab	8.37 ± 0.12 a	8.02 ± 0.14 a	32.27 ± 0.42 ab
Non-Bt	2	7.86 ± 0.13 b	7.56 ± 0.13 a	7.64 ± 0.12 a	36.29 ± 0.75 a
Homozygous Bt	2	7.54 ± 0.22 b	8.06 ± 0.14 a	8.41 ± 0.13 a	46.78 ± 0.42 a
Heterozygous Bt	2	7.47 ± 0.12 b	7.69 ± 0.15 a	7.69 ± 0.09 ab	40.53 ± 0.66 a
Non-Bt	4	7.62 ± 0.18 b	8.49 ± 0.27 a	8.27 ± 0.24 a	28.12 ± 0.65 b
Homozygous Bt	4	8.31 ± 0.11 ab	7.46 ± 0.11 ab	8.31 ± 0.30 a	34.31 ± 0.24 ab
Heterozygous Bt	4	7.96 ± 0.26 b	7.36 ± 0.35 ab	8.17 ± 0.11 a	31.29 ± 0.26 ab
Non-Bt	8	9.78 ± 0.23 a	7.80 ± 0.31 a	7.46 ± 0.15 a	22.35 ± 0.87 b
Homozygous Bt	8	9.04 ± 0.21 a	6.98 ± 0.24 ab	7.49 ± 0.28 ab	26.14 ± 0.43 b
Heterozygous Bt	8	8.51 ± 0.26 ab	7.05 ± 0.26 ab	8.55 ± 0.13 a	25.49 ± 0.53 b

**Table 7:** Physicochemical properties of stock leachates from different rice types. Note: Values are presented as means ± standard errors (SE); different lowercase letters within the same parameter indicate significant differences among treatments (*p* < 0.05).

Parameter	Non-Bt	Homozygous Bt	Heterozygous Bt
Bt protein (ng·mL^−1^)	—	1405.21 ± 77.21 a	1159.42 ± 38.97 b
Total N (mg·L^−1^)	11.23 ± 0.35 a	10.63 ± 0.19 a	10.54 ± 0.38 a
Total P (mg·L^−1^)	1.46 ± 0.02 b	2.24 ± 0.03 a	1.25 ± 0.04 c
Total K (mg·L^−1^)	22.86 ± 0.23 c	26.36 ± 0.32 a	24.46 ± 0.43 b

## Data Availability

The data presented in this study are available upon request from the corresponding author.

## Supplementary Materials

The following supporting information can be downloaded at: https://www.mdpi.com/article/10.3390/ijms27219962/s1.

## References

[1] Zhang S.Y., Lin C.Y., Ye Y.X., Wang Z., Chen M.X., Liu Q., Peng Y.F., Li Y.H., Huang F.N. (2023). Insect-resistant and herbicide-tolerant rice carrying cry1Ab-vip3Af2 and cp4-epsps genes. Acta Agric. Zhejiangensis.

[2] Wang H., Cao Z.H., Ye G.Y., Chen M.X., Liu Q., Peng Y.F. (2023). Effects of common rice disease and insect stresses on Bt protein expression in cry1C-transgenic insect-resistant rice. J. Biosaf..

[3] Chen X.P., Wang J.M., Zhu H.J., Li Y.H., Zhang Y., Liu B. (2013). Research progress on the effects of Bt-transgenic crops on aquatic ecosystems. Chin. J. Appl. Environ. Biol..

[4] Carstens K., Anderson J., Bachman P., De Schrijver A., Dively G., Federici B., Hamer M., Gielkens M., Jensen P., Lamp W. (2012). Genetically modified crops and aquatic ecosystems: Considerations for environmental risk assessment and non-target organism testing. Transgenic Res..

[5] Liu Y., Li J., Luo Z., Wang H., Liu F. (2016). The fate of fusion Cry1Ab/1Ac proteins from Bt-transgenic rice in soil and water. Ecotoxicol. Environ. Saf..

[6] Wang Y., Hu H., Huang J., Li J., Liu B., Zhang G. (2013). Determination of the movement and persistence of Cry1Ab/1Ac protein released from Bt transgenic rice under field and hydroponic conditions. Soil Biol. Biochem..

[7] Cellini F., Chesson A., Colquhoun I., Constable A., Davies H., Engel K., Gatehouse A., Kärenlampi S., Kok E., Leguay J.-J. (2004). Unintended effects and their detection in genetically modified crops. Food Chem. Toxicol..

[8] Wang S. (2021). Effects of Straw Returning on Seed Germination and Maize Seedling Growth. Master’s Dissertation.

[9] Gao Y.J. (2019). Safety Evaluation of Two Bt Proteins and Cry1C-transgenic Rice to Zebrafish. Master’s Thesis.

[10] Zhang L., Guo R.Q., Fang Z.X., Liu B. (2016). Genetically modified rice Bt-Shanyou63 expressing Cry1Ab/c protein does not harm *Daphnia magna*. Ecotoxicol. Environ. Saf..

[11] Chen Y. (2017). Ecological Safety Assessment of cry1C-Transgenic Rice to *Daphnia magna *Ph.D. Thesis. Thesis.

[12] Zhang L., Han J., Liu B. (2018). Ecotoxicological effects of cry1Ab and epsps-transgenic maize C0030.3.5 on *Daphnia magna*. Asian J. Ecotoxicol..

[13] Mo S.Y., Liu Y.F., Sun L.C., Zhang Y., Li Y.H., Chen X.P. (2016). Effects of Cry1Ab/Ac-transgenic rice on zooplankton species diversity in paddy fields. Chin. J. Ecol..

[14] Li G., Wang Y., Liu B., Zhang G. (2014). Transgenic *Bacillus thuringiensis* (Bt) rice is safer to aquatic ecosystems than its non-transgenic counterpart. PLoS ONE.

[15] Meissle M., Naranjo S.E., Romeis J. (2025). Meta-Analysis on Effects of Bt-Maize on Nontarget Invertebrates—Data Transportability across Continents. Plants People Planet.

[16] Wu N., Shi W.C., Liu W.W., Gao Z., Han L.Z., Wang X.F. (2021). Differential Impact of Bt-Transgenic Rice Plantings on Bacterial Community in Three Niches over Consecutive Years. Ecotoxicol. Environ. Saf..

[17] Deng J.X., Wang Y.M., Yang F.Y., Liu Y., Liu B. (2019). Persistence of insecticidal Cry toxins in Bt rice residues under field conditions estimated by biological and immunological assays. Sci. Total Environ..

[18] Liu L., Wu L.H., Knauth S., Eickhorst T. (2019). Degradation of transgenic Bt-rice straw incorporated with two different paddy soils. J. Environ. Manag..

[19] Nguyen T.-D., Huynh T.-N., Nguyen V.-T., Dinh K.V., Wiegand C., Pham T.L., Bui M.-H., Itayama T., Tran N.T., Wang Z. (2025). Multigenerational testing reveals delayed chronic toxicity of bisphenol A to *Daphnia magna*: A common characteristic of endocrine-disrupting chemicals?. Environ. Pollut..

[20] Shim S.B., Yoon M.H., An H.J. (2025). Microplastic fiber-induced transgenerational epigenetic disruption impairs fitness in *Daphnia magna*. Aquat. Toxicol..

[21] Griffiths N.A., Tank J.L., Royer T.V., Rosi E.J., Shogren A.J., Frauendorf T.C., Whiles M.R. (2017). Occurrence, leaching, and degradation of Cry1Ab protein from transgenic maize detritus in agricultural streams. Sci. Total Environ..

[22] Chen Y., Yang Y., Zhu H., Romeis J., Li Y., Peng Y., Chen X. (2018). Safety of *Bacillus thuringiensis* Cry1C protein for *Daphnia magna* based on different functional traits. Ecotoxicol. Environ. Saf..

[23] Chen Y., Gao Y., Zhu H., Romeis J., Li Y., Peng Y., Chen X. (2018). Effects of straw leachates from Cry1C-expressing transgenic rice on the development and reproduction of *Daphnia magna*. Ecotoxicol. Environ. Saf..

[24] Chen Y., Romeis J., Meissle M. (2021). Addressing the Challenges of Non-Target Feeding Studies with Genetically Engineered Plant Material—Stacked Bt Maize and *Daphnia magna*. Ecotoxicol. Environ. Saf..

[25] Chen Y., Romeis J., Meissle M. (2021). Performance of *Daphnia magna* on Flour, Leaves, and Pollen from Different Maize Lines: Implications for Risk Assessment of Genetically Engineered Crops. Ecotoxicol. Environ. Saf..

[26] Li S.H., Liu K., Zha W.J., Xu H., Li P., Zhou L., You A. (2021). Effects of BPH9- and Bar-transgenic brown planthopper-resistant and herbicide-tolerant rice ‘H23’ on non-target organisms. Biodivers. Sci..

[27] Zhang L., Shen W.J., Fang Z.X., Liu B. (2019). Effects of transgenic maize ZZM030 on growth and reproduction of *Daphnia magna*. J. Agro-Environ. Sci..

[28] Lv B., Zhou Y., Peng Y.-D., Wang Z., Song Q.-S. (2022). Integrative analysis identifies the safety of transgenic Cry1Ab rice to non-target spider *Pardosa pseudoannulata*. Biol. Control.

[29] Eladel H., Battah M., Dawa A., Abd-Elhay R., Anees D. (2019). Effect of rice straw extracts on growth of two phytoplankton isolated from a fish pond. J. Appl. Phycol..

[30] Wang J., Chen X., Li Y., Su C., Ding J., Peng Y. (2014). Green algae (*Chlorella pyrenoidosa*) adsorbs *Bacillus thuringiensis* (Bt) toxin, Cry1Ca insecticidal protein, without an effect on growth. Ecotoxicol. Environ. Saf..

[31] Bøhn T., Primicerio R., Hessen D.O., Traavik T. (2008). Reduced fitness of *Daphnia magna* fed a Bt-transgenic maize variety. Arch. Environ. Contam. Toxicol..

[32] Bøhn T., Traavik T., Primicerio R. (2010). Demographic responses of *Daphnia magna* fed transgenic Bt-maize. Ecotoxicology.

[33] Bøhn T., Rover C.M., Semenchuk P. (2016). *Daphnia magna* negatively affected by chronic exposure to purified Cry-toxins. Food Chem. Toxicol..

[34] Cuhra M., Traavik T., Bøhn T. (2015). Life cycle fitness differences in *Daphnia magna* fed Roundup-Ready soybean or conventional soybean or organic soybean. Aquac. Nutr..

[35] Holderbaum D.F., Cuhra M., Wickson F., Orth A.I., Nodari R.O., Bøhn T. (2015). Chronic responses of *Daphnia magna* under dietary exposure to leaves of a transgenic (Event MON810) Bt-maize hybrid and its conventional near-isoline. J. Toxicol. Environ. Health.

[36] EFSA (2010). Guidance on the environmental risk assessment of genetically modified plants. EFSA J..

[37] Romeis J., Raybould A., Bigler F., Candolfi M.P., Hellmich R.L., Huesing J.E., Shelton A.M. (2013). Deriving criteria to select arthropod species for laboratory tests to assess the ecological risks from cultivating arthropod-resistant genetically engineered crops. Chemosphere.

[38] Gao Y., Huang C., Han L., Xie J., Chen X. (2025). Effects of genetically modified rice expressing Cry1C protein on zebrafish after a 90-day dietary intake. Sci. Rep..

[39] OECD (2012). Test No. 211: *Daphnia magna* Reproduction Test. OECD Guidelines for the Testing of Chemicals, Section 2.

[40] Esterhuizen M., Monticelli M., Lee S.A., Kim Y., Pflugmacher S., Kim Y.J. (2024). Oxidative stress status and antioxidative responses in neonate versus adult *Daphnia magna* exposed to polystyrene leachate. Toxicol. Environ. Health Sci..

[41] Jeremias G., Muñiz-González A.-B., Gonçalves F.J.M., Martínez-Guitarte J.-L., Asselman J., Pereira J.L. (2024). History of exposure to copper influences transgenerational gene expression responses in *Daphnia magna*. Epigenetics.

[42] Zhang C., Suo W., Pan Y., Feng Y. (2025). Effects of Bt Rice Straw Extract on Seed Germination and Plant Growth of Pakchoi: Novel Variables of Cropping System. Plants.

[43] Chen Y., Tang H., Ma S., Lin X., Guo J., Jia R., Duan Y., Yang Y. (2026). Plant background, not Bt proteins, drives *Daphnia magna* life-table responses: A multi-tissue chronic study with multi-Cry Bt maize. Front. Plant Sci..

[44] Gao M., Zhang Z., Lv M., Song W., Lv Y. (2018). Toxic effects of nanomaterial-adsorbed cadmium on *Daphnia magna*. Ecotoxicol. Environ. Saf..

[45] Welch B.L. (1947). The generalization of “Student’s” problem when several different population variances are involved. Biometrika.

[46] Holm S. (1979). A simple sequentially rejective multiple test procedure. Scand. J. Stat..

[47] Duncan D.B. (1955). Multiple range and multiple F tests. Biometrics.

[48] Agbangba C.E., Aide E.S., Honfo H., Kakai R.G. (2024). On the use of post-hoc tests in environmental and biological sciences: A critical review. Heliyon.

[49] Shapiro S.S., Wilk M.B. (1965). An analysis of variance test for normality (complete samples). Biometrika.

[50] Brown M.B., Forsythe A.B. (1974). Robust tests for the equality of variances. J. Am. Stat. Assoc..

[51] Agresti A. (2002). Categorical Data Analysis.

