# Supplementary material for: Effects of Bt Rice Straw Leachates on Daphnia magna
Source: Int J Mol Sci. 2026 Nov 6;27(21):9962. doi: 10.3390/ijms27219962 (PMC13764512; doi:10.3390/ijms27219962)
Supplement: Supplementary file 1 [file ijms-27-09962-s001.zip › ijms-4646486-supplementary.pdf]

Supplementary Materials: Statistical Analysis Results (Tables S1–S9)

Note: ANOVA results are presented as degrees of freedom, F statistics, and P values. P values for comparisons at the same concentration were adjusted using the Holm method.

Table S1. Two-way ANOVA of biochemical endpoints in two generations of Daphnia magna

Generation	Biochemical endpoint	Effect	Degrees of freedom (df_1, df_2)	F statistic	P value
G1	CAT	Straw type	2,36	52.311	2.23e-11
G1	CAT	Leachate concentration	3,36	10.323	4.81e-05
G1	CAT	Straw type × concentration	6,36	40.323	1.54e-14
G1	POD	Straw type	2,36	74.021	1.76e-13
G1	POD	Leachate concentration	3,36	39.734	1.63e-11
G1	POD	Straw type × concentration	6,36	38.729	2.86e-14
G1	SOD	Straw type	2,36	45.809	1.28e-10
G1	SOD	Leachate concentration	3,36	119.613	8.86e-19
G1	SOD	Straw type × concentration	6,36	44.945	2.85e-15
G1	MDA	Straw type	2,36	4.364	0.0201
G1	MDA	Leachate concentration	3,36	12.980	6.69e-06
G1	MDA	Straw type × concentration	6,36	2.323	0.0535
G2	CAT	Straw type	2,36	24.728	1.75e-07
G2	CAT	Leachate concentration	3,36	816.988	3.78e-33
G2	CAT	Straw type × concentration	6,36	166.796	9.57e-25
G2	POD	Straw type	2,36	8.489	0.000954
G2	POD	Leachate concentration	3,36	9.276	0.000111
G2	POD	Straw type × concentration	6,36	9.530	2.81e-06
G2	SOD	Straw type	2,36	149.119	3.81e-18
G2	SOD	Leachate concentration	3,36	48.708	9.35e-13
G2	SOD	Straw type × concentration	6,36	20.209	3.48e-10
G2	MDA	Straw type	2,36	2.101	0.137
G2	MDA	Leachate concentration	3,36	2.730	0.0581
G2	MDA	Straw type × concentration	6,36	2.079	0.0801

Note: The generations were analyzed separately, with straw type, leachate concentration, and their interaction as factors. Four independent biological replicates were included per treatment.

Table S2. Model diagnostics and sensitivity analyses for the biochemical ANOVAs

Generation	Endpoint	Shapiro P	Brown–Forsythe P	Log Shapiro P	Log Brown–F P	Original interaction P	Log interaction P	HC3 interaction P
G1	CAT	1.30e-05	0.241	4.08e-06	0.242	1.54e-14	7.40e-14	3.52e-19
G1	POD	0.712	0.709	0.415	0.623	2.86e-14	2.41e-15	2.31e-12
G1	SOD	0.759	0.0314	0.605	0.0735	2.85e-15	7.05e-15	2.01e-15
G1	MDA	0.387	0.176	0.0869	0.272	0.0535	0.319	0.00705
G2	CAT	0.668	0.0354	0.362	0.0442	9.57e-25	6.75e-26	3.23e-26
G2	POD	5.52e-05	0.787	0.00225	0.868	2.81e-06	1.56e-07	2.80e-09
G2	SOD	0.000253	0.255	0.000444	0.401	3.48e-10	1.51e-08	1.31e-10
G2	MDA	2.99e-08	0.511	0.415	0.54	0.0801	0.0123	7.47e-06

Note: Model assumptions were assessed using the Shapiro–Wilk and Brown–Forsythe tests. Results from the original, log-transformed, and HC3-robust analyses were compared.

Table S3a. First-generation CAT activity: comparisons with concentration-matched non-Bt controls

Concentration (g/L)	Comparison with matched non-Bt control	P value
1	Homozygous Bt	0.00374
1	Heterozygous Bt	0.002
2	Homozygous Bt	0.000124
2	Heterozygous Bt	0.00147
4	Homozygous Bt	0.000124
4	Heterozygous Bt	0.569
8	Homozygous Bt	0.135
8	Heterozygous Bt	0.0159

Note: Two-sided Welch tests compared each Bt treatment with the non-Bt control at the same concentration. The eight comparisons for each endpoint were adjusted using the Holm method. The same applies to Tables S3a–S3h.

Table S3b. First-generation POD activity: comparisons with concentration-matched non-Bt controls

Concentration (g/L)	Comparison with matched non-Bt control	P value
1	Homozygous Bt	0.895
1	Heterozygous Bt	0.0311
2	Homozygous Bt	0.0256
2	Heterozygous Bt	0.0256
4	Homozygous Bt	0.496
4	Heterozygous Bt	0.00836
8	Homozygous Bt	0.000264
8	Heterozygous Bt	0.00206

Table S3c. First-generation SOD activity: comparisons with concentration-matched non-Bt controls

Concentration (g/L)	Comparison with matched non-Bt control	P value
1	Homozygous Bt	0.00128
1	Heterozygous Bt	0.00128
2	Homozygous Bt	0.262
2	Heterozygous Bt	0.00255
4	Homozygous Bt	0.799
4	Heterozygous Bt	0.391
8	Homozygous Bt	0.799
8	Heterozygous Bt	0.00681

Table S3d. First-generation MDA content: comparisons with concentration-matched non-Bt controls

Concentration (g/L)	Comparison with matched non-Bt control	P value
1	Homozygous Bt	1
1	Heterozygous Bt	1
2	Homozygous Bt	1
2	Heterozygous Bt	0.34
4	Homozygous Bt	0.00647
4	Heterozygous Bt	0.521
8	Homozygous Bt	1
8	Heterozygous Bt	1

Table S3e. Second-generation CAT activity: comparisons with concentration-matched non-Bt controls

Concentration (g/L)	Comparison with matched non-Bt control	P value
1	Homozygous Bt	0.000889
1	Heterozygous Bt	0.263
2	Homozygous Bt	0.00288
2	Heterozygous Bt	0.000443
4	Homozygous Bt	0.00181
4	Heterozygous Bt	0.00161
8	Homozygous Bt	0.000338
8	Heterozygous Bt	0.00288

Table S3f. Second-generation POD activity: comparisons with concentration-matched non-Bt controls

Concentration (g/L)	Comparison with matched non-Bt control	P value
1	Homozygous Bt	0.574
1	Heterozygous Bt	0.0878
2	Homozygous Bt	0.0411
2	Heterozygous Bt	0.0179
4	Homozygous Bt	0.000386
4	Heterozygous Bt	0.315
8	Homozygous Bt	0.0411
8	Heterozygous Bt	0.315

Table S3g. Second-generation SOD activity: comparisons with concentration-matched non-Bt controls

Concentration (g/L)	Comparison with matched non-Bt control	P value
1	Homozygous Bt	0.0503
1	Heterozygous Bt	0.133
2	Homozygous Bt	1
2	Heterozygous Bt	0.00313
4	Homozygous Bt	0.00313
4	Heterozygous Bt	0.0177
8	Homozygous Bt	1
8	Heterozygous Bt	0.00286

Table S3h. Second-generation MDA content: comparisons with concentration-matched non-Bt controls

Concentration (g/L)	Comparison with matched non-Bt control	P value
1	Homozygous Bt	1
1	Heterozygous Bt	0.25
2	Homozygous Bt	0.607
2	Heterozygous Bt	0.00891
4	Homozygous Bt	0.814
4	Heterozygous Bt	1
8	Homozygous Bt	1
8	Heterozygous Bt	1

Table S4. Fisher's exact tests of 21-day survival in two generations of Daphnia magna

Generation	Concentration (g/L)	Comparison	Bt survivors	Non-Bt survivors	Fisher P value
G1	1	Homozygous Bt vs non-Bt	40	40	1
G1	1	Heterozygous Bt vs non-Bt	40	40	1
G1	2	Homozygous Bt vs non-Bt	38	40	0.4937
G1	2	Heterozygous Bt vs non-Bt	39	40	1
G1	4	Homozygous Bt vs non-Bt	37	38	1
G1	4	Heterozygous Bt vs non-Bt	37	38	1
G1	8	Homozygous Bt vs non-Bt	35	36	1
G1	8	Heterozygous Bt vs non-Bt	36	36	1
G2	1	Homozygous Bt vs non-Bt	39	40	1
G2	1	Heterozygous Bt vs non-Bt	40	40	1
G2	2	Homozygous Bt vs non-Bt	38	40	0.4937
G2	2	Heterozygous Bt vs non-Bt	39	40	1
G2	4	Homozygous Bt vs non-Bt	37	39	0.6153
G2	4	Heterozygous Bt vs non-Bt	38	39	1
G2	8	Homozygous Bt vs non-Bt	36	37	1
G2	8	Heterozygous Bt vs non-Bt	36	37	1

Note: Forty D. magna were initially exposed per treatment. Two-sided Fisher's exact tests compared 21-day survival between Bt treatments and their concentration-matched non-Bt controls.

Table S5. ANOVA and pairwise comparisons of growth parameters in first-generation Daphnia magna

(A) Two-way ANOVA

Endpoint	Effect	Degrees of freedom (df_1, df_2)	F statistic	P value
Body length (mm)	Straw type	2, 72	7.694	0.0009
Body length (mm)	Leachate concentration	3, 72	19.316	2.72e-09
Body length (mm)	Straw type × concentration	6, 72	10.839	1.46e-08
Body width (mm)	Straw type	2, 72	7.380	0.0012
Body width (mm)	Leachate concentration	3, 72	4.019	0.0106
Body width (mm)	Straw type × concentration	6, 72	2.377	0.0376
Spine length (mm)	Straw type	2, 72	1.953	0.1493
Spine length (mm)	Leachate concentration	3, 72	8.708	5.29e-05
Spine length (mm)	Straw type × concentration	6, 72	0.682	0.6644
Body weight (mg)	Straw type	2, 72	3.857	0.0256

Endpoint	Effect	Degrees of freedom (df_1, df_2)	F statistic	P value
Body weight (mg)	Leachate concentration	3, 72	18.945	3.69e-09
Body weight (mg)	Straw type × concentration	6, 72	2.268	0.0463

(B) Comparisons with concentration-matched non-Bt controls

Endpoint	Concentration (g/L)	Comparison with matched non-Bt control	P value
Body length (mm)	1	Homozygous Bt	0.0735
Body length (mm)	1	Heterozygous Bt	0.8809
Body length (mm)	2	Homozygous Bt	0.0288
Body length (mm)	2	Heterozygous Bt	0.8946
Body length (mm)	4	Homozygous Bt	0.0032
Body length (mm)	4	Heterozygous Bt	0.0249
Body length (mm)	8	Homozygous Bt	0.0013
Body length (mm)	8	Heterozygous Bt	0.0168
Body width (mm)	1	Homozygous Bt	0.0358
Body width (mm)	1	Heterozygous Bt	0.9788
Body width (mm)	2	Homozygous Bt	0.9788
Body width (mm)	2	Heterozygous Bt	0.9788
Body width (mm)	4	Homozygous Bt	0.2821
Body width (mm)	4	Heterozygous Bt	0.0409
Body width (mm)	8	Homozygous Bt	0.9788
Body width (mm)	8	Heterozygous Bt	0.2821
Spine length (mm)	1	Homozygous Bt	1.0000
Spine length (mm)	1	Heterozygous Bt	1.0000
Spine length (mm)	2	Homozygous Bt	1.0000
Spine length (mm)	2	Heterozygous Bt	1.0000
Spine length (mm)	4	Homozygous Bt	0.5865
Spine length (mm)	4	Heterozygous Bt	1.0000
Spine length (mm)	8	Homozygous Bt	0.0328
Spine length (mm)	8	Heterozygous Bt	1.0000
Body weight (mg)	1	Homozygous Bt	0.2450
Body weight (mg)	1	Heterozygous Bt	1.0000

Endpoint	Concentration (g/L)	Comparison with matched non-Bt control	P value
Body weight (mg)	2	Homozygous Bt	1.0000
Body weight (mg)	2	Heterozygous Bt	0.3358
Body weight (mg)	4	Homozygous Bt	0.3358
Body weight (mg)	4	Heterozygous Bt	1.0000
Body weight (mg)	8	Homozygous Bt	1.0000
Body weight (mg)	8	Heterozygous Bt	0.1351

Note: Part A presents two-way ANOVA results; Part B presents comparisons between Bt treatments and non-Bt controls at the same concentration.

Table S6. ANOVA and pairwise comparisons of reproductive parameters in first-generation Daphnia magna

(A) Two-way ANOVA

Endpoint	Effect	Degrees of freedom (df_1, df_2)	F statistic	P value
Time to first reproduction (d)	Straw type	2, 468	39.509	1.4e-16
Time to first reproduction (d)	Leachate concentration	3, 468	20.598	1.49e-12
Time to first reproduction (d)	Straw type × concentration	6, 468	3.816	0.0010
First brood size	Straw type	2, 468	2.293	0.1021
First brood size	Leachate concentration	3, 468	1.789	0.1483
First brood size	Straw type × concentration	6, 468	15.659	2.07e-16
Number of molts	Straw type	2, 468	5.148	0.0061
Number of molts	Leachate concentration	3, 468	6.917	0.0001
Number of molts	Straw type × concentration	6, 468	9.061	2.19e-09
Total offspring	Straw type	2, 468	120.851	4.85e-43
Total offspring	Leachate concentration	3, 468	265.543	1.31e-100
Total offspring	Straw type × concentration	6, 468	14.224	6.55e-15

(B) Comparisons with concentration-matched non-Bt controls

Endpoint	Concentration (g/L)	Comparison with matched non-Bt control	P value
Time to first reproduction (d)	1	Homozygous Bt	0.0007
Time to first reproduction (d)	1	Heterozygous Bt	0.0046
Time to first reproduction (d)	2	Homozygous Bt	0.0008
Time to first reproduction (d)	2	Heterozygous Bt	8.57e-11
Time to first reproduction (d)	4	Homozygous Bt	0.3686
Time to first reproduction (d)	4	Heterozygous Bt	0.0575

Endpoint	Concentration (g/L)	Comparison with matched non-Bt control	P value
Time to first reproduction (d)	8	Homozygous Bt	0.0575
Time to first reproduction (d)	8	Heterozygous Bt	1.42e-07
First brood size	1	Homozygous Bt	1.88e-07
First brood size	1	Heterozygous Bt	1.19e-10
First brood size	2	Homozygous Bt	0.0185
First brood size	2	Heterozygous Bt	0.5810
First brood size	4	Homozygous Bt	0.0001
First brood size	4	Heterozygous Bt	0.0013
First brood size	8	Homozygous Bt	0.0011
First brood size	8	Heterozygous Bt	0.5810
Number of molts	1	Homozygous Bt	0.0021
Number of molts	1	Heterozygous Bt	0.3860
Number of molts	2	Homozygous Bt	0.4259
Number of molts	2	Heterozygous Bt	0.0083
Number of molts	4	Homozygous Bt	0.0083
Number of molts	4	Heterozygous Bt	0.0133
Number of molts	8	Homozygous Bt	0.4259
Number of molts	8	Heterozygous Bt	0.0097
Total offspring	1	Homozygous Bt	2.16e-14
Total offspring	1	Heterozygous Bt	3.21e-20
Total offspring	2	Homozygous Bt	9.43e-20
Total offspring	2	Heterozygous Bt	1.97e-05
Total offspring	4	Homozygous Bt	1.19e-08
Total offspring	4	Heterozygous Bt	1.19e-08
Total offspring	8	Homozygous Bt	0.7816
Total offspring	8	Heterozygous Bt	0.0092

Note: Part A presents two-way ANOVA results; Part B presents comparisons between Bt treatments and non-Bt controls at the same concentration.

Table S7. ANOVA and pairwise comparisons of growth parameters in second-generation Daphnia magna

(A) Two-way ANOVA

Endpoint	Effect	Degrees of freedom (df_1, df_2)	F statistic	P value

Endpoint	Effect	Degrees of freedom (df_1, df_2)	F statistic	P value
Body length (mm)	Straw type	2, 72	1.594	0.2102
Body length (mm)	Leachate concentration	3, 72	31.504	4.06e-13
Body length (mm)	Straw type × concentration	6, 72	8.692	4.05e-07
Body width (mm)	Straw type	2, 72	1.978	0.1458
Body width (mm)	Leachate concentration	3, 72	9.820	1.65e-05
Body width (mm)	Straw type × concentration	6, 72	1.667	0.1417
Spine length (mm)	Straw type	2, 72	1.542	0.2210
Spine length (mm)	Leachate concentration	3, 72	5.693	0.0015
Spine length (mm)	Straw type × concentration	6, 72	0.444	0.8469
Body weight (mg)	Straw type	2, 72	2.484	0.0905
Body weight (mg)	Leachate concentration	3, 72	5.703	0.0015
Body weight (mg)	Straw type × concentration	6, 72	1.726	0.1271

(B) Comparisons with concentration-matched non-Bt controls

Endpoint	Concentration (g/L)	Comparison with matched non-Bt control	P value
Body length (mm)	1	Homozygous Bt	0.1750
Body length (mm)	1	Heterozygous Bt	1.0000
Body length (mm)	2	Homozygous Bt	0.0432
Body length (mm)	2	Heterozygous Bt	1.0000
Body length (mm)	4	Homozygous Bt	0.0036
Body length (mm)	4	Heterozygous Bt	0.0348
Body length (mm)	8	Homozygous Bt	1.0000
Body length (mm)	8	Heterozygous Bt	0.5030
Body width (mm)	1	Homozygous Bt	0.3478
Body width (mm)	1	Heterozygous Bt	1.0000
Body width (mm)	2	Homozygous Bt	1.0000
Body width (mm)	2	Heterozygous Bt	0.0219
Body width (mm)	4	Homozygous Bt	1.0000
Body width (mm)	4	Heterozygous Bt	1.0000
Body width (mm)	8	Homozygous Bt	1.0000
Body width (mm)	8	Heterozygous Bt	1.0000
Spine length (mm)	1	Homozygous Bt	1.0000

Endpoint	Concentration (g/L)	Comparison with matched non-Bt control	P value
Spine length (mm)	1	Heterozygous Bt	1.0000
Spine length (mm)	2	Homozygous Bt	0.8635
Spine length (mm)	2	Heterozygous Bt	1.0000
Spine length (mm)	4	Homozygous Bt	1.0000
Spine length (mm)	4	Heterozygous Bt	1.0000
Spine length (mm)	8	Homozygous Bt	1.0000
Spine length (mm)	8	Heterozygous Bt	1.0000
Body weight (mg)	1	Homozygous Bt	1.0000
Body weight (mg)	1	Heterozygous Bt	1.0000
Body weight (mg)	2	Homozygous Bt	1.0000
Body weight (mg)	2	Heterozygous Bt	1.0000
Body weight (mg)	4	Homozygous Bt	0.3930
Body weight (mg)	4	Heterozygous Bt	1.0000
Body weight (mg)	8	Homozygous Bt	0.2099
Body weight (mg)	8	Heterozygous Bt	1.0000

Note: Part A presents two-way ANOVA results; Part B presents comparisons between Bt treatments and non-Bt controls at the same concentration.

Table S8. ANOVA and pairwise comparisons of reproductive parameters in second-generation Daphnia magna

(A) Two-way ANOVA

Endpoint	Effect	Degrees of freedom (df_1, df_2)	F statistic	P value
Time to first reproduction (d)	Straw type	2, 468	7.385	0.0007
Time to first reproduction (d)	Leachate concentration	3, 468	37.155	1.49e-21
Time to first reproduction (d)	Straw type × concentration	6, 468	5.025	5.24e-05
First brood size	Straw type	2, 468	0.164	0.8491
First brood size	Leachate concentration	3, 468	3.443	0.0167
First brood size	Straw type × concentration	6, 468	11.137	1.25e-11
Number of molts	Straw type	2, 468	5.624	0.0039
Number of molts	Leachate concentration	3, 468	4.494	0.0040
Number of molts	Straw type × concentration	6, 468	4.702	0.0001
Total offspring	Straw type	2, 468	131.800	3.96e-46
Total offspring	Leachate concentration	3, 468	482.634	8.7e-143
Total offspring	Straw type × concentration	6, 468	9.312	1.17e-09

(B) Comparisons with concentration-matched non-Bt controls

Endpoint	Concentration (g/L)	Comparison with matched non-Bt control	P value
Time to first reproduction (d)	1	Homozygous Bt	0.0030
Time to first reproduction (d)	1	Heterozygous Bt	0.0007
Time to first reproduction (d)	2	Homozygous Bt	0.4302
Time to first reproduction (d)	2	Heterozygous Bt	0.0914
Time to first reproduction (d)	4	Homozygous Bt	0.0086
Time to first reproduction (d)	4	Heterozygous Bt	0.4302
Time to first reproduction (d)	8	Homozygous Bt	0.0799
Time to first reproduction (d)	8	Heterozygous Bt	0.0030
First brood size	1	Homozygous Bt	7.57e-06
First brood size	1	Heterozygous Bt	2.49e-09
First brood size	2	Homozygous Bt	0.0533
First brood size	2	Heterozygous Bt	0.5145
First brood size	4	Homozygous Bt	0.0053
First brood size	4	Heterozygous Bt	0.0533
First brood size	8	Homozygous Bt	0.1198
First brood size	8	Heterozygous Bt	0.1354
Number of molts	1	Homozygous Bt	0.7109
Number of molts	1	Heterozygous Bt	0.0593
Number of molts	2	Homozygous Bt	0.0003
Number of molts	2	Heterozygous Bt	1.0000
Number of molts	4	Homozygous Bt	1.0000
Number of molts	4	Heterozygous Bt	1.0000
Number of molts	8	Homozygous Bt	1.0000
Number of molts	8	Heterozygous Bt	4.01e-06
Total offspring	1	Homozygous Bt	4.11e-09
Total offspring	1	Heterozygous Bt	0.0002
Total offspring	2	Homozygous Bt	3.81e-17
Total offspring	2	Heterozygous Bt	0.0002
Total offspring	4	Homozygous Bt	4.79e-11
Total offspring	4	Heterozygous Bt	0.0002

Endpoint	Concentration (g/L)	Comparison with matched non-Bt control	P value
Total offspring	8	Homozygous Bt	0.0005
Total offspring	8	Heterozygous Bt	0.0030

Note: Part A presents two-way ANOVA results; Part B presents comparisons between Bt treatments and non-Bt controls at the same concentration.

Table S9. Cry1Ab Protein Measurement Results

Generation	Rice type	Concentration (g/L)	Measurement 1	Measurement 2	Mean
G1	Non-Bt	1	0.9626	0.9218	0.9422
G1	Non-Bt	2	0.8543	0.9075	0.8809
G1	Non-Bt	4	0.9271	0.8969	0.912
G1	Non-Bt	8	0.8791	0.8774	0.8782
G1	Homozygous Bt	1	0.8614	0.8454	0.8534
G1	Homozygous Bt	2	0.8347	0.8596	0.8472
G1	Homozygous Bt	4	0.8489	0.8365	0.8427
G1	Homozygous Bt	8	0.8756	0.8738	0.8747
G1	Heterozygous Bt	1	0.7868	0.8081	0.7974
G1	Heterozygous Bt	2	0.9022	0.9377	0.92
G1	Heterozygous Bt	4	0.9093	0.9022	0.9058
G1	Heterozygous Bt	8	0.8418	0.8489	0.8454
G2	Non-Bt	1	0.6021	0.5932	0.5976
G2	Non-Bt	2	0.8774	0.8738	0.8756
G2	Non-Bt	4	0.5506	0.5488	0.5497
G2	Non-Bt	8	0.6767	0.6749	0.6758
G2	Homozygous Bt	1	0.6767	0.6749	0.6758
G2	Homozygous Bt	2	0.8259	0.8241	0.825
G2	Homozygous Bt	4	0.6749	0.6678	0.6713
G2	Homozygous Bt	8	0.6784	0.6696	0.674
G2	Heterozygous Bt	1	0.5612	0.6163	0.5888
G2	Heterozygous Bt	2	0.6447	0.6305	0.6376
G2	Heterozygous Bt	4	0.3978	0.3676	0.3827
G2	Heterozygous Bt	8	0.5168	0.6074	0.5621

Note: The two measurements for each treatment were technical replicates.
